# BH4 supplementation reduces retinal cell death in ischaemic retinopathy

**DOI:** 10.1038/s41598-023-48167-5

**Published:** 2023-12-02

**Authors:** Kevin S. Edgar, Ciara Cunning, Tom A. Gardiner, Denise M. McDonald

**Affiliations:** 1 Wellcome-Wolfson Institute for Experimental Medicine, Queen’s University Belfast, Belfast, 97 Lisburn Road, BT9 7BL UK; 2 School of Medicine, Dentistry and Biomedical Sciences, Centre for Biomedical Sciences Education, Queen’s University Belfast, Belfast, UK

**Keywords:** Retinopathy of prematurity, Retina

## Abstract

Dysregulation of nitric oxide (NO) production can cause ischaemic retinal injury and result in blindness. How this dysregulation occurs is poorly understood but thought to be due to an impairment in NO synthase function (NOS) and nitro-oxidative stress. Here we investigated the possibility of correcting this defective NOS activity by supplementation with the cofactor tetrahydrobiopterin, BH_4_. Retinal ischaemia was examined using the oxygen-induced retinopathy model and BH_4_ deficient Hph-1 mice used to establish the relationship between NOS activity and BH_4_. Mice were treated with the stable BH_4_ precursor sepiapterin at the onset of hypoxia and their retinas assessed 48 h later. HPLC analysis confirmed elevated BH_4_ levels in all sepiapterin supplemented groups and increased NOS activity. Sepiapterin treatment caused a significant decrease in neuronal cell death in the inner nuclear layer that was most notable in WT animals and was associated with significantly diminished superoxide and local peroxynitrite formation. Interestingly, sepiapterin also increased inflammatory cytokine levels but not microglia cell number. BH_4_ supplementation by sepiapterin improved both redox state and neuronal survival during retinal ischaemia, in spite of a paradoxical increase in inflammatory cytokines. This implicates nitro-oxidative stress in retinal neurones as the cytotoxic element in ischaemia, rather than enhanced pro-inflammatory signalling.

## Introduction

Nitric oxide has multiple roles in the retina and is essential for normal tissue function and survival. For example, in the retinal vasculature it is responsible for maintaining vasomotor function and promotes VEGF-induced angiogenesis, while in neural cells it is required for neuromodulation of synaptic activity, neurovascular coupling, and retinal ganglion-photoreceptor cell connectivity and is also involved in light perception^1–4^. Additionally, in inflammatory cells it promotes infection clearance and tissue regeneration after injury^5^. These diverse functions are carried out by the three NO producing enzymes, the nitric oxide synthases (NOS): endothelial NOS (eNOS/NOS-3), neuronal NOS (nNOS/NOS-1) and inducible NOS (iNOS/NOS-2), which are expressed in endothelial, neuronal and immune cells respectively. All three isoforms are mechanistically similar and produce NO by catalyzing the oxidation of l-arginine to form citrulline and NO. The most significant difference between the isoforms is that eNOS and nNOS are constitutively expressed and regulated by changes in intracellular calcium whereas iNOS is usually only expressed in pathological situations such as inflammation or hypoxia. In addition, when expressed, calcium-calmodulin binds automatically to iNOS producing an active enzyme capable of releasing high amounts of NO, which are much higher than that produced by the other two isoforms. All three isoforms are also regulated in a cell type specific manner, dependent on multiple post-translational modifications and their physical interaction with binding partners that modulate their activity.

**Figure 1:**
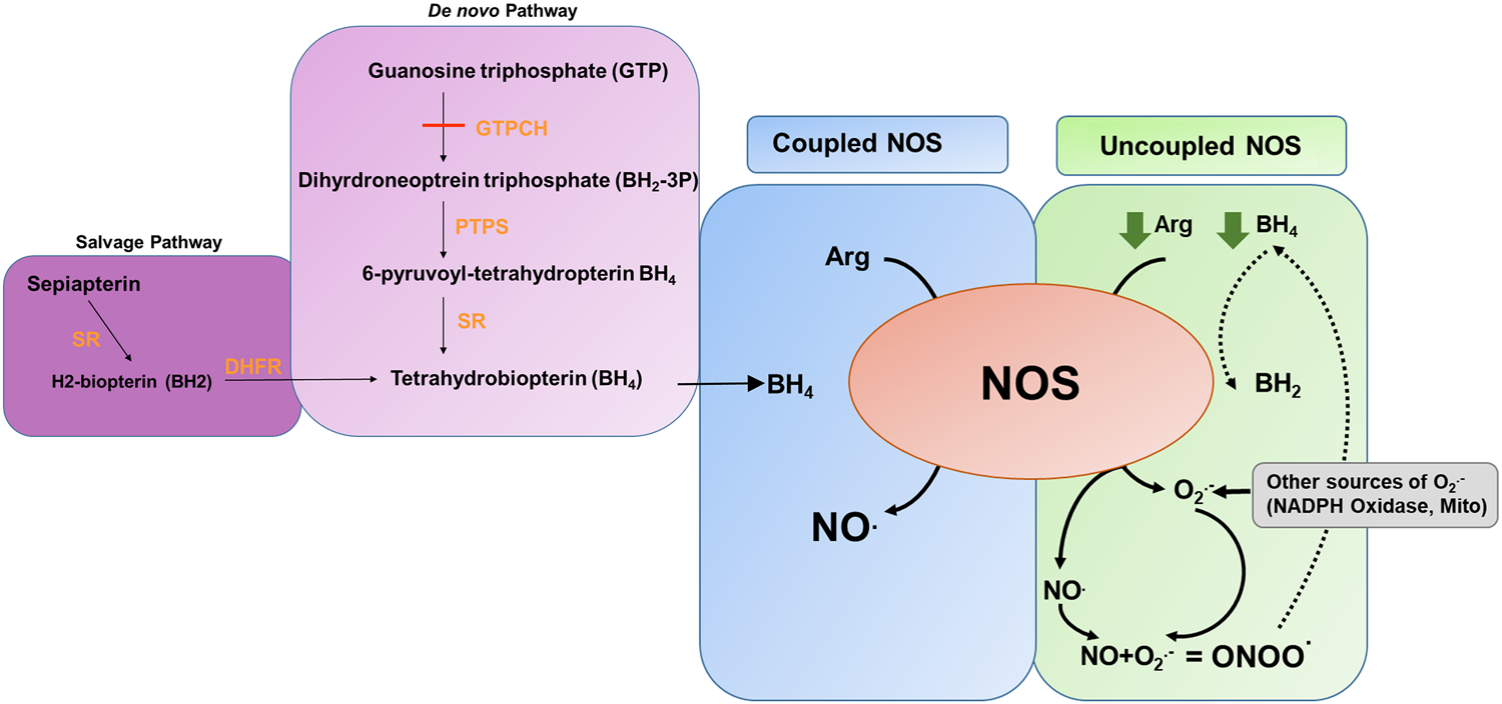
Biochemical pathway showing the production of BH_4_ from GTP or sepaipterin and the impact on NOS function in the absence and presence of oxidative stress. BH_4_ is produced from GTP by the de novo pathway by a series of enzymatic steps where GTPCH is the rate limiting step (red line) that determines the rate of BH_4_ production. Alternatively, BH_4_ can be produced from sepiapterin by the salvage pathway. BH_4_ in sufficient amounts promotes the production of NO from coupled NOS (blue panel). When the supply of BH_4_ is insufficient NOS is uncoupled and produces superoxide (O_2_^−^) as well as NO (green panel). The reaction of NO and O_2_^−^ produces peroxynitrire (ONOO^−^) which causes the further oxidation of BH_4_ (dotted line). NO can also be scavenged by O_2_^−^ produced from other sources such as the NADPH oxidase or mitrochondria. *GTPCH* GTP cyclohydrolase, *PTPS* 6-pyruvoyltetrahydropterin synthase, *SR* sepaipterin reducatase, *DHFR* dihydrofolate reductase.

The importance of the NOS enzymes is particularly apparent in disease when their function is compromised. For example, dysregulated eNOS is associated with impaired vascular growth and similarly, deregulated nNOS may cause neural cell death in response to ischaemia^6–10^. This often occurs in the presence of reactive oxygen species (ROS) which quenches NO, reducing its bioavailability (Fig. 1). In addition, the direct reaction of superoxide and NO produces peroxynitrite, a highly reactive and damaging free radical. Taken together such increased nitro-oxidative stress may precipitate cell death in retinal ischaemia^8,9,11–13^. Retinal ischaemia is a serious sight-threatening complication of several diseases including retinopathy of prematurity (ROP) and diabetic retinopathy, where it induces destructive retinal neovascularization. Both diseases are associated with oxidative or metabolic damage and degeneration of the blood vessels that supply the neural retina, leading to tissue hypoxia. This induces the activation of the hypoxia-inducible transcription factors HIF-1α and HIF-2α in neural and glial cells of the now avascular ischaemic inner retina leading to expression of pro-angiogenic growth factors, principally vascular endothelial growth factor (VEGFA)^14,15^. VEGFA stimulates angiogenesis in the vasculature adjacent to the ischaemic regions; however, the strength of the angiogenic stimulus results in disorganised exuberant growth of new vessels. Unfortunately, these vessels do not obey normal directional cues that would facilitate revascularisation of the ischaemic tissue, rather they escape the confines of the retina and proliferate in the pre-retinal area within the vitreous body^7,14,16,17^. This abnormal vascular growth fails to resolve the underlying ischaemia, but may cause tractional retinal detachment through introduction of contractile scar tissue to the vitreous body. Clinically, it has been shown that even a brief duration of ischaemia can have long lasting effects on neural function: for example, infants with ROP may have visual impairments that persist until adulthood^18–20^.

At the cellular level, studies using the oxygen-induced retinopathy model (OIR) in mice have demonstrated the effects of ischaemia in the central vessel free or avascular zone of the inner retina, causing hypoxia-induced cell death to cells residing there, consisting of bipolar, amacrine and horizontal neurons, and Müller glial cells^9,12^. Moreover defects in this inner nuclear region of the retina have also been shown to occur early in diabetic retinopathy which suggests a unique vulnerability of these cells to ischaemic or metabolic stress in diabetes or models of retinopathy of prematurity^21^. Mechanistically, the early ischaemic insult and cell death is associated with nitro-oxidative stress derived from two main sources: neural cell derived—nNOS and iNOS induced by retinal hypoxia in Müller cells and microglial cells (MG), the resident immune cells of the retina which we have previously found to be present in this avascular zone^7,9,12^. Interestingly, there are conflicting reports on the role of inflammatory cells such as MG in ischaemia either shown to promote disease progression or contribute to its resolution. Indeed, MG cells have been associated with increasing neurodegeneration and conversely also with the clearance of apoptotic cells and resolution of tissue damage in ischaemia^9,12,22,23^. These differences are due in part to the cell’s ability to switch function in response to environmental cues: pro-inflammatory, characterised by a high nitric oxide (NO)/ROS/reactive nitrogen species (RNS) output, or anti-inflammatory, characterised by a low NO/ROS/RNS output and associated with aiding tissue repair. MG are also highly motile therefore differences in their localisation or the time point at which they are analysed may also lead to conflicting hypotheses for their role in disease. For example, blood vessel associated MG are proposed to promote neovascular growth whereas we have shown that MG localised to the inner nuclear layer (INL), where ischaemic damage is most marked, are engaged in and associated with removal of apoptotic cells^12,24^. These conflicting roles indicate a need to have a better appreciation of the role of MG in individual stages of disease.

Current therapies focus on late disease and strategies are aimed at removing the neovascular tufts rather than protecting the neural retina from damage. Currently, there are no treatments that specifically target the neural retina in DR and ROP and all treatment options focus on later more advanced neovascular stages of disease. Therefore, finding ways to protect the neural retina from ischaemic injury would be therapeutically beneficial. In contrast to the late vascular phase characterised by aberrant neovascularisation (NV), the mechanisms that lead to neural damage are less well understood. Thus, here our aim was to investigate the possibility of protecting neuronal cells in ischaemia and in order to achieve this we focused on the possibility of correcting NOS function.

All NOS isoforms are dependent on the co-factor tetrahydrobiopterin (BH_4_) for the synthesis of NO which is necessary to facilitate the flow of electrons or reducing equivalents from the reductase to the oxidase domain of the enzyme. When BH_4_ levels are insufficient the reductase and oxidase functions become disconnected or uncoupled and activated oxygen or superoxide leaves the active site of the enzyme leading to superoxide production instead of NO which leads to reduced NO availability and elevated oxidative stress^8,25–32^. Enhancing BH_4_ has been shown to reverse this effect, therefore, here our aim was to investigate the impact of BH_4_ on protecting the retina from ischaemic damage. We have previously demonstrated that supplementing BH_4_ using a stable precursor, sepiapterin, during the hyperoxic vaso-obliterative phase of murine oxygen-induced retinopathy (OIR), can correct NOS dysfunction and protect the vasculature from oxidative stress^6,33^. Accordingly, here we hypothesised that BH_4_ supplementation during the second ischaemic phase of the OIR model may also be protective to the retina by focusing on its impact on the two cell types most likely to be affected by NOS dysfunction, neural cells of the inner retina and MG. The level of endogenous BH_4_ is determined by guanosine triphosphate cyclohydrolase-1(GTPCH), the rate-limiting enzyme controlling the synthesis of this cofactor (Fig. 1). Therefore, here we used hyperphenylalaninemic (Hph-1) mice, deficient in GTPCH with the heterozygotes and homozygote animals producing different BH_4_ levels to demonstrate the relationship between BH_4_ and NOS output, and sepiapterin supplementation to investigate the impact of an improved BH_4_ supply in the ischaemic phase of the murine OIR model. We present novel findings showing that BH_4_ supplementation, through treatment with its precursor sepiapterin, is neuroprotective in the retina during ischaemic hypoxia by increasing NO and decreasing nitro-oxidative species with normalization of the cellular redox state. Interestingly, this neuroprotective effect occurred in the presence of increased inflammatory cell markers suggesting that the cytotoxic component of hypoxia is the generation of peroxynitrite when NOS is present and that MG provide a supportive or pro-resolving role in ischaemia.

## Results

### Ischaemia induces cell death in the avascular neural retina in phase II of OIR: effect on Hph-1 phenotype

**Figure 2:**
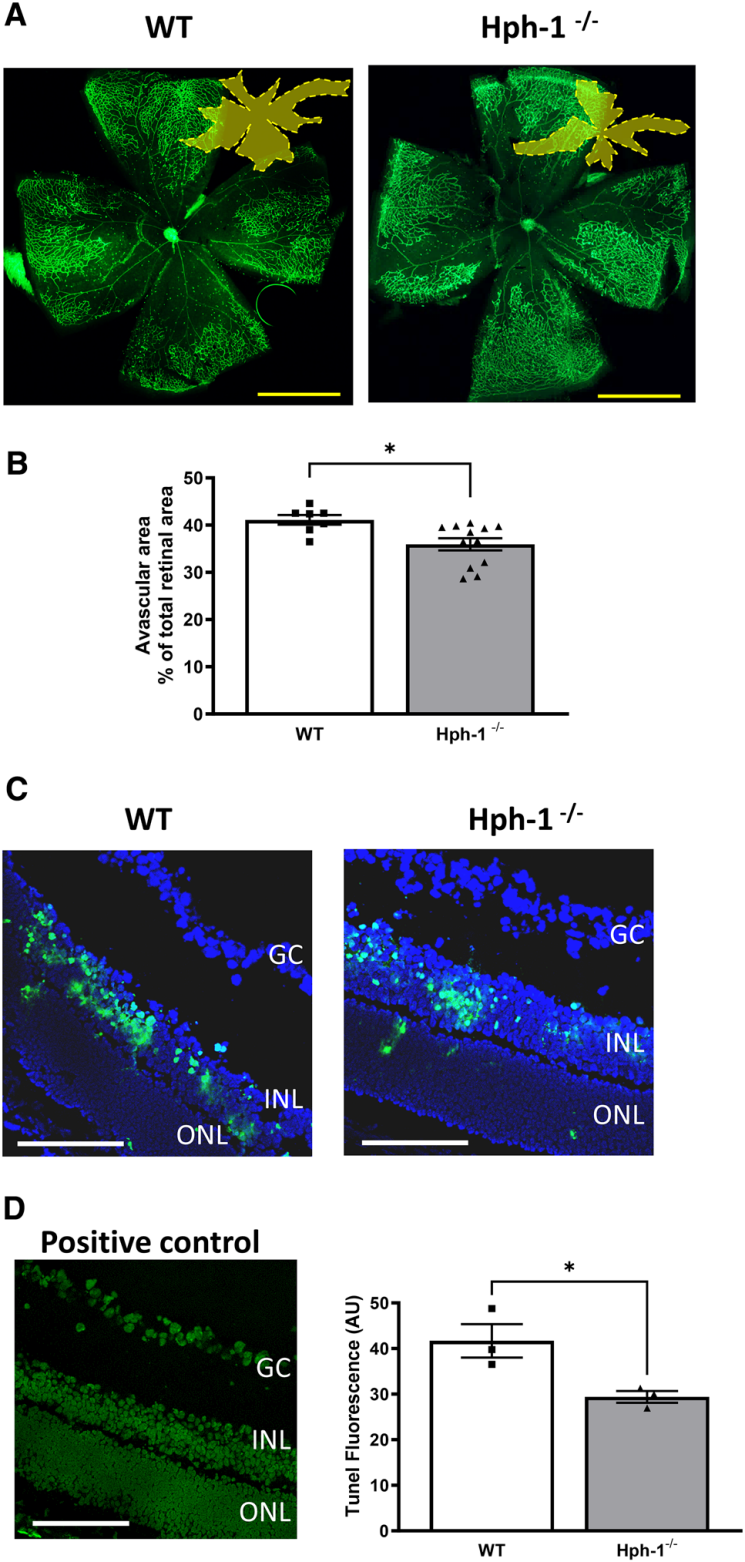
Reduced apoptotic damage in the avascular zone of P13 OIR mice from Hph-1^−/−^ mice compared to WT controls. (**A**) Representative images of WT and Hph-1^−/−^ P13 OIR flat-mounts were stained with isolectin B4 to visualise the retinal vessels (green labelling) and confirm vascular obliteration in the pre-hypoxic phase (P7–12). (**B**) Using Image J software, total vascular and avascular areas (dark central area and yellow masking in inset) were demarcated and measured as a percentage of the total retinal area. There are significantly greater blood vessels remaining from Hph-1^−/−^ mice than WT, *P < 0.05, n ≥ 8 per group from 6 litters. Scale bars are 500 µm. (**C**) Apoptosis was measured using TUNEL staining in retinal sections 24 h following hypoxia (P13 in the OIR model).  Sections for TUNEL were taken from and focused on the central vessel free zone seen in (**A**). Example images from WT and Hph-1^−/−^ retinal sections showing TUNEL positive cells (green) and DAPI nuclear staining (blue). Positive control image is also shown in (**D**). (**D**) Quantification of TUNEL fluorescence showed significantly less TUNEL positive cells in the inner nuclear layer of retinal sections from Hph-1^−/−^ mice, *P < 0.05, n = 3 animals from different litters, for each animal eight retinal sections were analysed and three non-overlapping images were taken per section. *GC* Ganglion cell layer, *INL* inner nuclear layer, *ONL* outer nuclear layer. Scale bars are 100 µm.

Acute retinal ischaemia 24 to 48 h following oxygen-induced loss of blood vessels and onset of hypoxia in the central retina has previously been shown to induce a marked increase in apoptotic cell death in the neural retina^9,12^. Accordingly, at P13, 24 h after the onset of hypoxia upon re-entry to room air conditions, we compared the extent of cell damage in Hph-1 and WT animals (Fig. 2). Vascular closure induced by hyperoxia exposure between P9 and P12 was initially confirmed by visualizing lectin positive blood vessel staining which showed the expected area of vaso-obliteration in the central retina (dark central zone in retinal images shown in Fig. 2A). We have previously shown that Hph-1 neonates have a genotype related adaption resulting in a reduced area of vascular closure following hyperoxia which is related to their higher retinal VEGF levels than their WT littermate controls^34^. This genotype-related difference was also evident in the retinas analysed at P13 in the present work and in accordance with our previous results at P12, the vascular coverage in the Hph-1 groups was marginally higher than in the WT animals (Fig. 2A,B). Notably, the vascularized areas in all groups were similar to those we previously reported for P12 in the OIR model, which typically shows little change in the central avascular area between P12 and P13. Next the level of apoptosis was measured using TUNEL staining of retinal sections taken through the central avascular region (Fig. 2C,D). In both WT and Hph-1 groups the majority of TUNEL positivity was seen in the INL and demonstrated the characteristic condensed chromatin and irregular nuclear morphology of cells undergoing apoptotic cell death. In addition, the location of the nuclei of the TUNEL positive cells, at the inner and central regions of the INL suggested that the majority of the dying cells were bipolar and amacrine neurons. Comparing the numbers of TUNEL stained cells across the genotypes examined showed that there was a significant reduction in TUNEL positive cells in Hph-1 mice compared to WT littermate controls (Fig. 2D).

### NOS activity and oxidative stress are reduced in Hph-1 animals at P13

**Figure 3:**
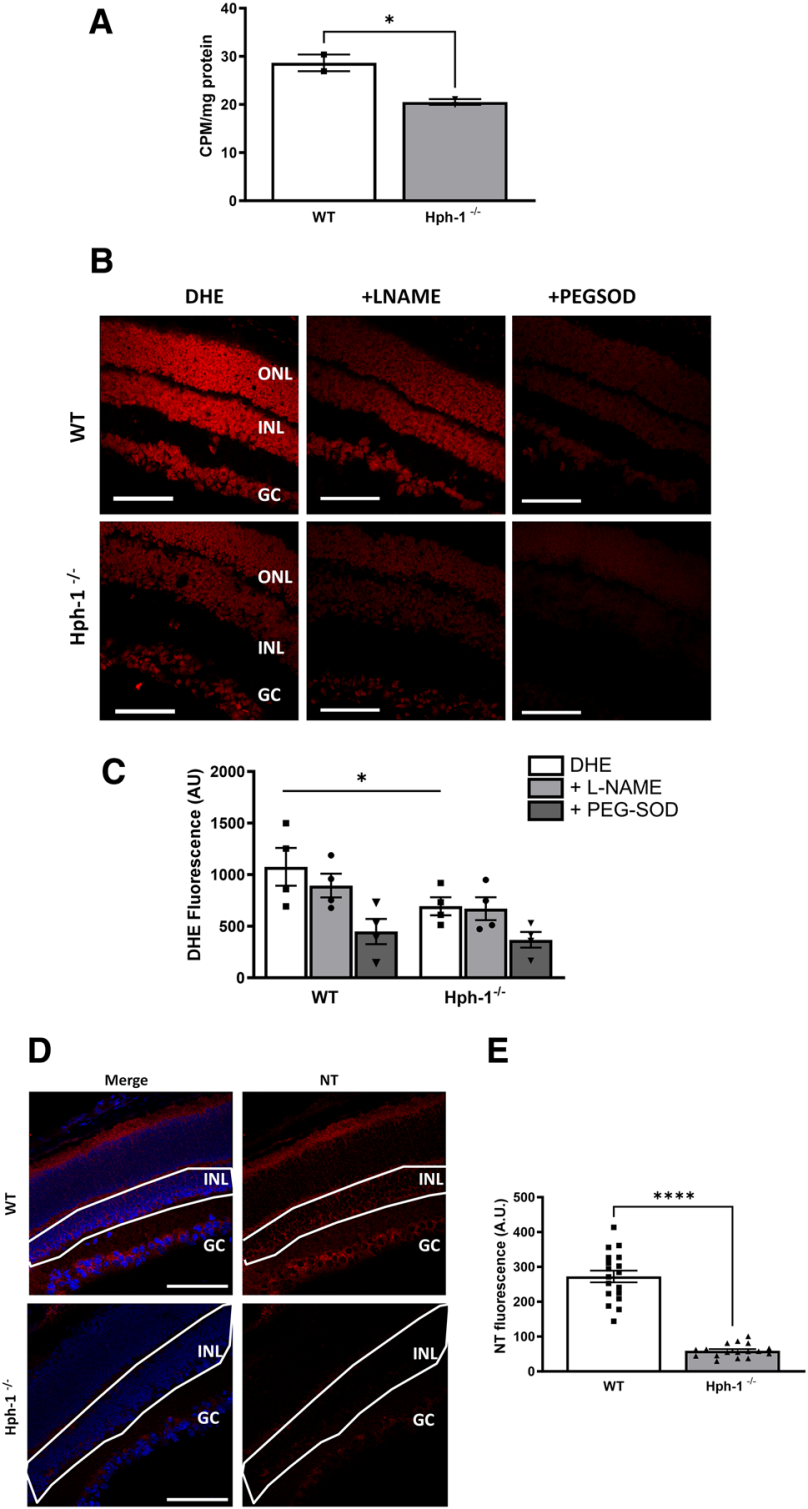
NOS activity, superoxide and nitrotryosine levels are reduced in P13 Hph-1^−/−^ mice compared to WT controls. (**A**) NOS activity is reduced in P13 Hph-1^−/−^ retinas compared to WT controls. NOS activity was determined by the conversion of radio-labelled arginine to citrulline using a NOS activity assay kit. NOS activity was significantly decreased in retinal samples from Hph-1^−/−^ mice compared to WT, consistent with the observed decrease in BH_4_ levels (*CPM* counts per minute). (**B**) Reduced superoxide generation in Hph-1^−/−^ retinal sections at P13. Superoxide was detected in retinal sections using the oxidative fluorescent dye DHE, with addition of inhibitors L-NAME and PEGSOD in sequential sections and quantified as described (**C**). (**D**) Nitrotyrosine (NT) immunoreactivity was used as an indicator of peroxynitrite formation. (**E**) NT fluorescence intensity in the inner nuclear layer (outlined area) was measured in retinal sections from mice following OIR at P13. There was significantly greater NT localised to the INL and the GC of WT retinal sections than Hph-1^−/−^ retinal sections. *ONL* outer nuclear layer, *INL* inner nuclear layer, *GC* ganglion cell layer, ***P < 0.01, *P < 0.05. For NOS activity—n = 4 retinas pooled were used from independent litters and assayed in triplicate. For DHE: n = 4 animals from different litters—for each animal 8 non-overlapping images were analysed. For NT n = 3 animals from different litters, for each animal 6 retinal sections were analysed and three images were taken per section. The quantification data for NT is representative of one independent experiment and the other two shown in Fig. S1. Scale bars are 100 µm.

NOS activity was measured in tissue collected at P13 and showed a decrease in total NOS activity when comparing WT and Hph-1^−/−^ animals (Fig. 3A) corresponding to a decrease in the ability of these animals to produce BH_4_. This incremental decrease in NOS activity, measured as the conversion of radiolabelled arginine to citrulline, from WT to Hph-1 mice correlates with the expected decrease in the ability of Hph-1 animals to produce tetrahydrobiopterin (BH_4_). Notably, additional BH_4_ was not added to the reaction buffer, therefore NOS activity was dependent on endogenous BH_4_ , allowing an accurate determination of in vivo NOS function. Dihydroethidium (DHE) was used to localise reactive oxygen species production in-situ in fresh-frozen tissue sections in the presence and absence of inhibitors which are added to the tissue samples ex vivo^12^. This assay measures the presence and activity of ROS producing enzymes at the time of tissue collection. Sections directly adjacent to those used for the TUNEL staining and from the same ischaemic region where used. In addition, the observed decrease in NO levels correlated with a decrease in ROS and RNS levels quantified as dihydroethidium (DHE) fluorescence (Fig. 3B,C) and nitrotyrosine (NT) immunostaining respectively (Fig. 3D,E, Fig. S1). This decrease was in line with the need for NO to produce peroxynitrite and RNS. The highest levels of NT staining were evident in the INL, close to the area showing most TUNEL positive staining, and the GCL. Treatment with L-NAME resulted in a marginal reduction in DHE fluorescence indicating NOS as source of superoxide (Fig. 3B,C). PEGSOD also decreased DHE florescence indicating additional enzymatic sources of superoxide production, such as the NADPH oxidases or mitochondria, contributing to the total oxidative status in the retina.

### The Hph-1 genotype shows enhanced numbers of MG co-localising with TUNEL positive apoptotic cells in the ischaemic retina

**Figure 4:**
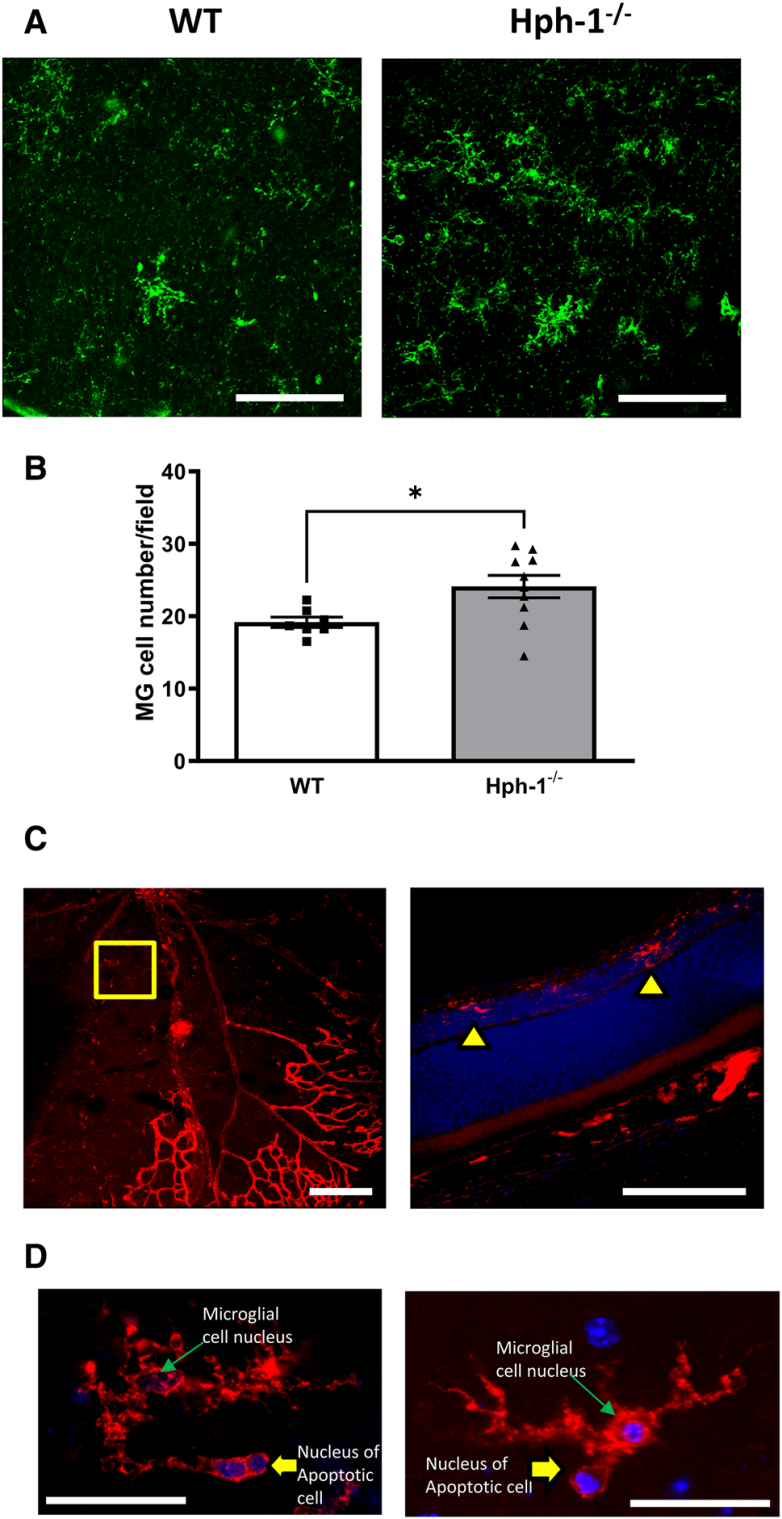
Retinal microglia cell numbers are higher in Hph-1^−/−^ mice in P13 retinas correlating with lower ischaemia-induced TUNEL positive staining and retinal cell death. The number of MG in retinas from WT and Hph-1 mice were quantified in full thickness z-stack images from lectin stained flat mounted retina. Notably there were significantly more MG cells in retinas from Hph-1^−/−^ animals compared to WT at P13 in ischaemic regions of the retina. (**A**) Representative images of MG cells in retinal flat mounts. MG are obvious from their lectin positivity and their morphological appearance. (**B**) Quantification of MG cell number in the INL, *P < 0.05, n ≥ 7 per group from 6 litters. Scale bars are 100 µm. (**C**) Low-power images of a retinal flat mount showing the avascular area where the z-series images shown in (**A**) were taken. The right hand panel in (**C**) shows a cross section of a flat mount taken in the avascular zone showing MG localised to the inner nuclear layer (yellow arrow). (**D**) High-power images from the ischaemic inner nuclear retina of the shows microglial cells phagocytosing DAPI positive apoptotic bodies (blue–yellow arrows). Scale bars are 100 µm.

Microglial (MG) cell density was estimated from GS isolectin B4 stained flat mounts from P13 animals and confocal microscopy imaging and cells identified by their positive staining with lectin and their characteristic morphology^12,35,36^. Notably, their very typical starshaped or dendritic morphology is easily identifiable in flat-mount preparations. In addition, since their processes span a number of retinal layers quantification of multi-layered confocal z-stack images were recorded through the full thickness of the inner retina in the ischaemic regions. The position of these cells in the INL was confirmed by z-stack confocal images and by sections taken from retinas post-flat mount preparation (Fig. 4). Following 24 h of hypoxia, retinal sections from the central avascular retina frequently showed microglia in the INL (Fig. 4A–D) rather than their normal perivasculature locations in the plexiform layers typically seen in the mature retina. Morphologically, the cells resembled activated cells with short retracted extensions rather than a dendritic appearance of cells typically seen in cells residing in the inner and outer plexiform layers. The localisation of microglia to the INL of avascular sections corresponded with the location of the greatest number of TUNEL positive cells. High power confocal images of MG cells in this region showed that they were engaged in phagocytosing apoptotic bodies (Fig. 4D). In addition, the numbers of MG cells in this region were increased in the Hph-1 animals compared to WT (Fig. 4A,B) which together with evidence of decreased TUNEL positivity suggests a pro-resolving role for MG in the ischaemic retina.

### Tyrosine hydroxylase immunostaining in the retina of Hph-1 mice at P7 shows evidence of a development delay in dendritic arborisation of amacrine cells and correlation with altered expression of neuronal differentiation markers

Retinal sections were immunostained for tyrosine hydroxylase (TH), a BH4 dependent enzyme that is necessary for dopamine production in amacrine cells. These cells localise in a very typical pattern at the innermost aspect of the INL (Fig. 3SA). TH immunopositive amacrine cells are the major dopaminergic cells in the retina and possess a number of processes that develop progressively from the early postnatal period to adulthood. The majority of TH-immunoreactive processes were shown to extend laterally into the distal portion of the IPL (Fig. 3SA). TH staining in P7 flat-mounts of Hph-1 mice (Fig. 3SA,B) was used to quantify the extent of TH positive dendrite extension. Visually, the TH positive amacrine cells were sparsely distributed and characterized by a plexus of processes extending from their cell bodies. There was no significant difference in the number of TH positive cell bodies between WT and Hph-1 mice. Consistent with the pattern observed in retinal sections, developing mice had significantly fewer TH positive processes extending from the TH positive cell bodies. Quantification of P7 dendritic processes showed a significant difference between TH staining in the P7 groups. Confocal z-stack images of the IPL and INL were taken of P7 retinal flat-mounts. P7 retinas displayed little overlap from adjacent cell processes and were quantified via the grid method as described in Fig. S2. Results show that TH immunopositive processes cover a significantly smaller area in P7 Hph-1^−/−^ retinas compared to WT littermates. TH positive processes in the adult retina were in vast abundance compared to the developing retina (Fig.2) and appeared as full developed pericellular rings. Gene expression in the retina of Hph-1 mice was carried out on each of the genotypes at P7 (Fig. S3C). As expected *Gch1* expression was decreased in Hph-1 genotypes. In addition, Hph-1s showed evidence of altered expression of well known neural differentiation markers such as *Notch1* and *Sox17 *proving further evidence of differences in maturation between the genotypes.

### Supplementation with sepiapterin between P12 and P14 OIR increases tissue BH_4_ levels

Previously we have shown that lung and brain tissue show similar elevated BH_4_ levels in response to sepiapterin treatment as those quantified in retinal samples^6^. In addition, here we show further evidence of a correlation between tissue BH_4_ levels between lung, brain and retinal samples following supplementation of P7 animals in a separate C57Bl6 wild type strain (Fig. S4A–C). These findings showed that this effect is consistent across several strains and that elevated BH_4_ levels are measurable up to 48 h post injection at P9. Therefore, here BH_4_ levels were measured in lung and brain to confirm the deficiency in Hph-1 mice and the ability of sepiapterin to increase BH_4_ at the tissue-level. HPLC analysis of mouse lung and brain tissue confirmed significantly lower tissue levels of BH_4_ in Hph-1^+/−^ and Hph-1^−/−^ compared to WT animals (Fig. S5A,B, Table S1). Supplementation of all genotypes with sepiapterin at P12 after hyperoxia resulted in an increase in lung and brain BH_4_ at P14 (Fig. S5A,B) compared to the corresponding vehicle control (VC) groups. This effect was most marked in lung samples (Fig. S5A) compared to brain tissue extracts (Fig. S5B). BH_2_, an oxidation product of BH_4_, showed similar incremental differences in levels as those shown for BH_4_ resulting in BH_4_/BH_2_ ratios that indicated preservation of BH_4_ levels (Fig. S5C,D), again most notably in lung samples. Whilst this was also found in brain tissue samples (Fig. S5E,F), the BH_4_/BH_2_ ratios were not as pronounced indicating some oxidation of BH_4_. BH_4_ levels have previously been shown to be lower in brain tissue compared to other tissues such as lung and kidney which may explain the lower BH_4_/BH_2_ ratios^37^.

### BH_4_ supplementation increases NOS activity and decreases DHE fluorescence, and nitrotyrosine (NT) adduction indicating reduced oxidative stress

**Figure 5:**
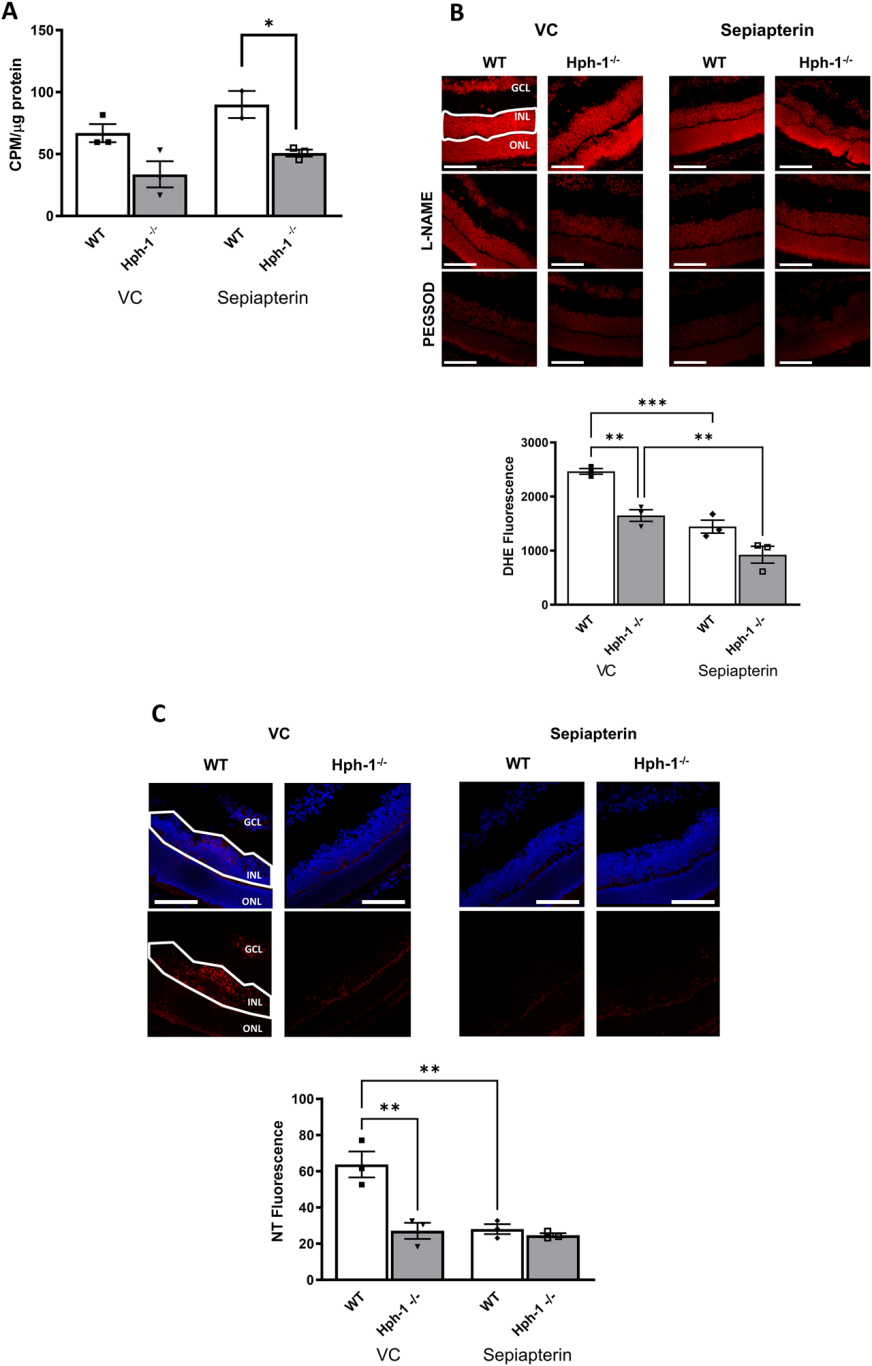
Nitric oxide synthase (NOS) activity is increased and oxidative stress decreased following BH_4_ supplementation. (**A**) Nitric oxide synthase (NOS) activity following BH_4_ supplementation. NOS activity was measured in P14 OIR retinal tissue with and without BH_4_ supplementation. NOS activity decreased across genotypes, consistent with the observed decrease in BH_4_ levels, NOS activity increased in all genotypes when supplemented with BH_4_ (*CPM* counts per minute). (**B**) Effect of BH_4_ supplementation on oxidative stress markers. Sections were incubated with dihydroethidium (DHE) in the presence or absence of L-NAME or PEGSOD and the fluorescence of the INL quantified. There was a reduction in the red fluorescence intensity in the sepiapterin treated sections indicating that supplementation of BH_4_ decreases the level of O_2_^−^ formed. The reaction was reduced further in sections treated with PEGSOD and L-NAME. Summary graph excluding inhibitors for easier visualisation of the difference between the genotypes and sepiapterin treated and untreated groups. (**C**) NT immunoreactivity following BH_4_ supplementation. P14 OIR retinal sections were incubated with an anti-NT antibody and NT immunoreactivity was measured in the INL of retinal sections from WT and Hph-1 mice following BH_4_ supplementation. The same confocal settings were maintained throughout imaging. WT mice showed the most marked decrease in NT expression following treatment with sepiapterin. Hph-1^−/−^ mice showed no further decrease following supplementation. NOS activity—n = 3 retinas pooled and assayed in triplicate. DHE: n = 3 animals from different litters—for each animal 8 non-overlapping images were analysed. For NT n = 3 animals from different litters—for each animal 6 retinal sections were analysed and three images were taken per section. Scale bars are 100 µm.

Following BH_4_ supplementation NOS activity increased in all genotypes with the increase in the WT group being the most significant. NOS activity in P14 OIR retinal tissue with and without BH_4_ supplementation showed levels of NO production from WT through to Hph-1^−/−^ (Fig. 5A) consistent with their differential BH_4_ quantities. NOS activity was 66.9 cpm/µg protein in WT and reduced to 33.6 in Hph-1^−/−^ mice. Following sepiapterin supplementation these values increased to 90.0 and 50.8 cpm/µg protein for WT and Hph-1^−/−^ respectively. Oxidative stress from superoxide production was determined by DHE in sections from all groups in the presence or absence of the inhibitors L-NAME or PEGSOD and the fluorescence quantified by automated image analysis. Sepiapterin treated groups had significantly diminished DHE fluorescence with the WT and Hph-1 groups showing significant decreases in DHE fluorescence of approximately 40% (Fig. 5B). This indicates that sepiapterin treatment decreases superoxide production, a reaction that was further reduced with PEGSOD and L-NAME incubation. NT fluorescence was also measured in the INL of retinal sections (Fig. 5C). Following OIR, the INL experiences the most cell death thus this layer may be more ischaemic than the ganglion cell layer or outer retina. In the untreated animals, NT fluorescence was significantly reduced in the Hph-1 retinas compared to WT controls. Following sepiapterin supplementation the wildtype group showed a significant decrease in NT expression. Hph-1 mice displayed little or no change following BH_4_ supplementation (Fig. 5C). Notably, taken together the upregulated NOS activity following BH4 supplementation with sepiapterin, and the decrease in superoxide and NT suggests an improvement in the tissue NO:O_2_^−^ ratio and lower nitro-oxidative stress.

### BH_4_ supplementation reduces ischaemia-induced apoptosis in the neural retina

**Figure 6:**
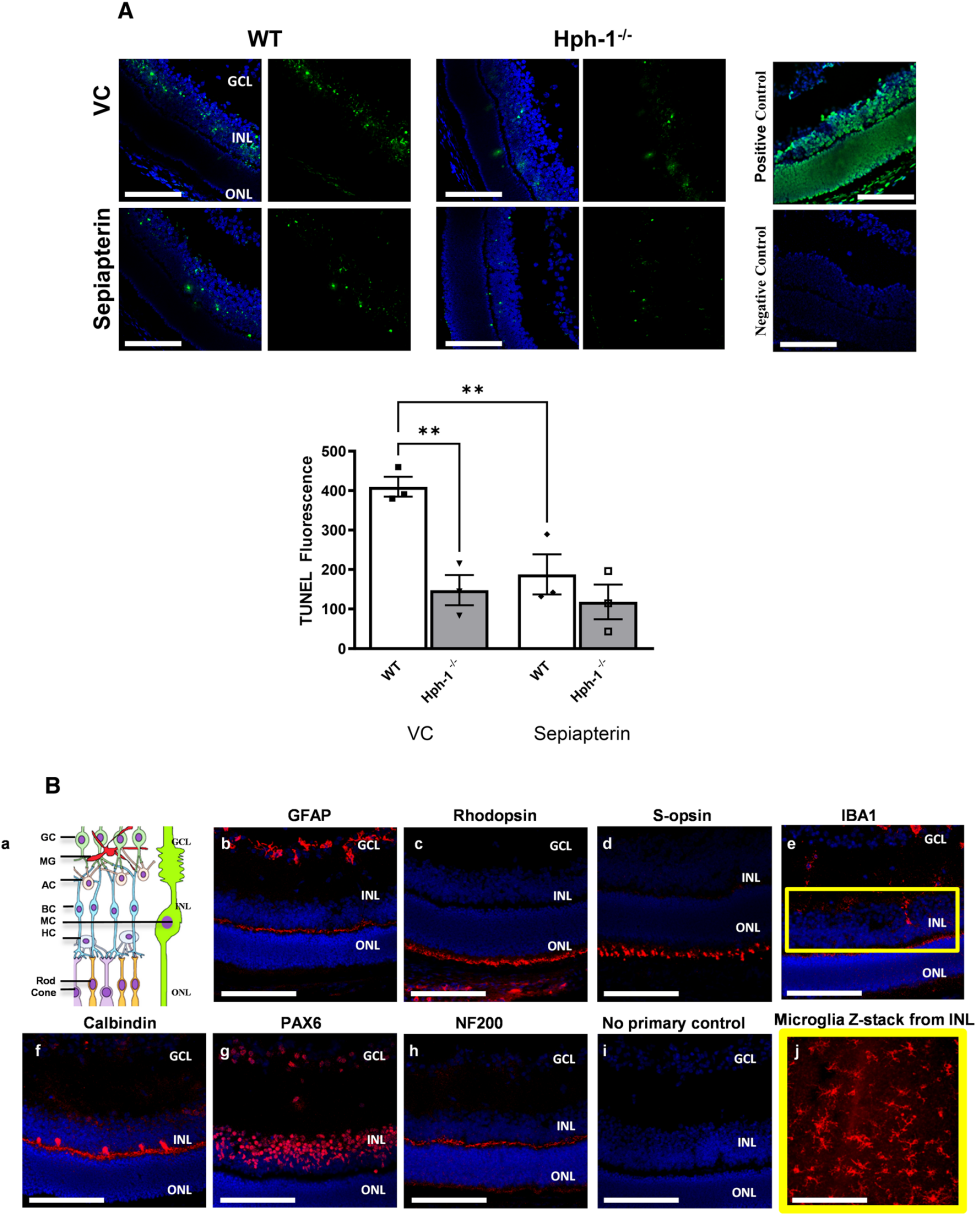
(**A**) BH_4_ supplementation reduces apoptosis in the ischaemic retina in phase 2 of the OIR model. Retinal cross sections from P14 WT and Hph-1^−/−^ mice show TUNEL-positive cells (green) within the inner nuclear layer. DAPI (blue) stains the nuclei. An example section treated with DNAase as a positive control and one receiving only labelling solution as a negative control. BH_4_ supplementation significantly decreased the level of apoptosis in the INL following ischaemia. (**B**) Investigating which cells are apoptotic in the INL. (**a**) Schematic diagram representing the main cells found in the GCL, INL and ONL. P14 WT OIR mouse retinal sections from the avascular area where stained for (**b**) GFAP (astrocytes), (**c**) rhodopsin (rod photoreceptors), (**d**) S-opsin (cones), (**e**) IBA1 (microglia), (**f**) calbindin (horizontal cells), (**g**) Pax6 (amacrine, ganglion and retinal progenitor cells) and (**h**) NF200 (neurofilament). (**i**) A control section was incubated without any primary antibody. (**j**) A z-stack from the INL of an isolectin stained retinal flatmount shows microglial cells also present in this layer. n = 3 animals from different litters—for each animal 8 retinal sections were analysed and three images were taken per section. Scale bars are 100 µm. BC bipolar cell, AC amacrine cell, GC ganglion cell, HC horizontal cell, MG microglial cell,  MC Müller cell.

WT and Hph-1 mice at P14 (48 h after vascular occlusion) were investigated to examine the effect of sepiapterin on hypoxia-induced cell death. Sections adjacent to those described for the investigation of NT and ROS above were studied (Fig. 6A). Positive TUNEL staining was again mainly located in the INL following ischaemia as evidenced by the green fluorescence and apoptotic nuclear morphology showing clearly identifiable shrunken nuclei with hypercondensed chromatin and nuclear fragments similar to those described in Fig. 2. TUNEL fluorescence decreased incrementally from WT through Hph-1^+/−^ and Hph-1^−/−^ (Fig. 6, Table S1) indicating a decrease in apoptosis between the genotypes. Comparison across the genotypes, showed that the largest number of TUNEL positive cells were in the WT control group and accordingly the biggest effect of sepiapterin supplementation was observed in this group, with a decrease of 54% (Fig. 6A bar chart). In contrast sepiapterin had less effect in the Hph-1 groups (31% in Hph-1^+/−^ (Table S1) and 20% in Hph-1^−/−^ animals), reflective of the lower levels of apoptosis observed at P13. Localisation of the MG showed their position in the INL, the site of maximal TUNEL staining (Fig. 6B).

### BH_4_ supplementation caused no significant change in MG numbers localising to the ischaemic retina, instead there was a significant enhancement in the expression of pro- and anti-inflammatory cytokines

MG showed an amoeboid morphology and were localised to the inner retinal layers corresponding to the locations of maximal TUNEL staining. Following supplementation there were no significant changes in the number of cells or their morphology from P13 and they all appeared similarly activated as evidenced by their short processes and bushy appearance (Fig. S6). Cytokines levels in VC and supplemented groups was determined using the Mouse Cytokine Array panel-A and analysed using tissue from heterozygote animals. Standard breeding protocols produced more heterozygotes (50%) than the WT and Hph-1^−/−^ animals therefore this tissue was used to determine the effects of sepiapterin supplementation on cytokine levels. Cytokine array showed a noticeable difference in the profile from sepiapterin treated retina compared to controls (Fig. S6). Notably, the sepiapterin treated retinae had a higher expression level for most of the cytokines assayed. Proinflammatory cytokines such as TNFα and IL-1α were significantly increased following sepiapterin supplementation as was soluble intercellular adhesion molecule-1 (sICAM-1), chemokine (C-X-C motif) ligand 1 (CXCL1/KC) and TIMP metallopeptidase inhibitor 1 (TIMP-1). Likewise, there was also a trend towards increased expression of IFN-γ and IL-1, following supplementation, differences in anti-inflammatory cytokines levels were less marked. Gene expression following BH_4_ supplementation by RT-PCR was also carried out on P14 OIR Hph-1 mice to analyse gene expression following BH_4_ supplementation. Heterozygote groups were used for comparisons of gene analysis and gene expression levels normalised to the housekeeping gene 18S (Fig. S7). A comparison of Ct values remained very similar when VC and sepiapterin samples were compared showing no effect of treatment on 18S expression. Gene expression comparison showed differences in expression levels of *Hif1α*, *Vegf*, and *Gch1* of between two to four fold following BH_4_ supplementation compared to the controls. *Mcp-1*(*Ccl2*), *Il1α* and *Il1β* were similarly increased which correlated with the increase in pro-inflammatory cytokines shown in the cytokine profiler results. *Arg*1 and *Arg*2 expression were increased by 1.4- and 1.3-fold. Macrophage inflammatory protein-2 (*Mip2*) (a functional murine homolog of *Il8* which is not expressed in mice) showed the largest increase in expression following BH_4_ supplementation with expression levels being 3.2-fold higher than control. Notably, this increase in inflammatory cytokine protein levels correlated to an increase in the expression of pro-inflammatory markers observed in the cytokine profiler array. This finding provided further evidence of an increase in total cytokine output and cells showing a pro-inflammatory phenotype in the BH_4_ treated retinas.

## Discussion

Ischaemic insult to the retina has serious consequences for sight. Most research has focused on the abnormal NV response to ischaemia that occurs late in disease and less on the impact on the neural retina which occurs immediately after the onset of hypoxia. Accordingly, currently there are no treatments that target this early pathology. Acute hypoxia causes cell death in the inner retina as a result of dysregulated nitric oxide (NO) production and elevated nitro-oxidative stress^7,9,12^. The reasons that nitro-oxidative stress occurs is poorly understood but is known to occur in the presence of low levels of the NOS cofactor BH_4_. Also, notably NOS dysfunction has been shown to be reversible in other disease settings^6,34^. Thus, here our aim was to investigate the effect of BH_4_ supplementation on ischaemia-induced neural damage.

Through this study we report several novel findings which together show that the bioavailability of the NOS cofactor BH_4_ has a profound effect on NOS activity, neuronal survival and redox status in the neural retina during the ischaemic phase of OIR. Initially, because of the previously reported developmental adaptions we noted in the retinas of Hph-1-GTPCH defective mice we investigated the impact of acute hypoxia in Hph-1 and WT littermate control mice at P13 after 24 h hypoxia^34^. Vascular area comparisons at P13 showed a small but significant increase in vascular coverage in the Hph-1 groups compared to WT animals as we previously found at P12 post hyperoxia. Next, since the avascular area of the retina has been shown to experience a high degree of hypoxia induced cell death, we focused on this zone to examine the extent of cell death in response to ischaemia^9,12^. All groups showed neural cell apoptosis in the avascular retina, located, almost exclusively, to the inner nuclear layer (INL) comprising amacrine, bipolar and Müller cells in response to OIR-induced ischaemia. Interestingly, in the Hph-1 animals the level of INL apoptosis was lower compared to the WT mice. This lower cell damage correlated to lower NOS activity levels in tissue extracts from Hph-1 mice, compared to WT littermates along with lower nitrotyrosine (NT) protein adduct levels, which was barely detectable in the ischaemic zone of the Hph-1 mice in agreement with the ability of Hph-1 mice to produce less NO. Indeed, it was interesting that the Hph-1 animals displayed lower levels of NT stress as it was also possible that their lower levels of BH_4_ could also have led to increased NOS dysfunction and associated ROS-induced apoptosis in these animals. Nevertheless, lower INL apoptosis is fully consistent with the undetectable level of peroxynitrite observed in Hph-1 mice in OIR in the present study. In light of these results, it appears that the modest NO generation in Hph-1 mice is insufficient to produce cytotoxic amounts of peroxynitrite.

Another possibility for the apparent paradoxical decrease in apoptosis in cells in the inner retina of the Hph-1 genotype was a result of differences in development as a result of lower BH4 levels. Indeed, previous work from our lab characterising the responsiveness of the Hph-1-GTPCH deficient mice to hyperoxia have shown that P7 and P12 Hph-1 mice have increased vascular density and experience increased vasoprotection in response to hyperoxic insult^34^. This finding correlated with higher neural cell numbers and GCL-localised VEGF levels compared to WT litter mate control mice indicating an effect or adaption that occurs during retinal development^34^. VEGF is a crucial survival factor for vascular cells and neurons, therefore, it is possible that the lower level of apoptosis at P13 in the Hph-1s is due to their higher retinal expression of this growth factor^38^. Moreover, BH_4_ is also a cofactor for the aromatic hydroxylases such as tyrosine hydroxylase (TH), the enzyme responsible for dopamine production, accordingly, Hph-1 animals produce lower dopamine levels. Dopamine acts as a negative regulator of VEGF thus the lower dopamine produced in Hph-1 animals would also remove this negative repression and enhance VEGFR2 signalling as it does in vascular endothelial cells to improve survival of retinal vessels^34,39^. Whilst this higher VEGF/VEGFR2 survival signalling may also potentially decrease hypoxia-induced apoptotic damage in retinal neurones we next sought to investigate the possibility that altered BH_4_ may have a direct impact on TH expression in amacrine cells, since these are localised to the inner nuclear region that suffers the greatest ischaemic insult. Amacrine cells are typically located to the upper aspect of the INL where they develop long dendritic projections that extend radially from postnatal day 5 to connect to other interneurons in the inner retina. In the current study we used their characteristic morphology and cell-specific TH immunoreactivity as markers so analyse possible differences in neural cell development in the Hph-1 retinas compared to controls. The number of TH immunopositive cell bodies was not significantly different in the retina of WT or Hph-1 in developing or adult mice. Instead, we found that at P7 the Hph-1 genotype had significantly less TH positive processes and reduced dendritic cell extensions compared to WT mice indicating a delay or dysregulation in maturation of amacrine cells. In adult retinas classical TH positive pericellular rings appeared similar across genotypes suggesting any alteration to the dopaminergic network in the Hph-1 animals may have partly resolved or may not have been as marked with increasing maturity. In agreement with the TH staining, transcriptome screening for typical factors involved in making cell fate decisions in retinal neurones, such as Notch, Dll4, Hey1 and Sox17 also showed a downregulation in their expression levels. Together this suggests that maturation of the inner retinal neural cell population is impacted by lower BH_4_ levels in Hph-1 animals. Interestingly, a similar defect or delay in neuronal maturation has been described in the human disease Dopa Responsive Dystonia (DRD), characterised by dopamine deficiency as a result of a germline mutation in one of the BH_4_ or TH processing enzymes such as GTPCH or sepiapterin reductase. In DRD, post mortem analysis of the dopaminergic cells of the substania nigra of affected individuals have been described as rounded and developmentally immature^40^. Moreover the observed defects in dendrite arborisation in the Hph-1 mice is similar to neuronal defects noted in induced pluripotent stem cells (iPSC) derived from patients with mutations in the TH gene^41^. iPSC derived neurones from these patients show abnormal morphology indicating an intrinsic defect in TH deficient neuronal cells similar to our observation in Hph-1 retinas. Similarly, *GCH* knockout embryos die in utero yet display no visible defects in organ morphology^42^. In summary, our findings suggest that the BH_4_ and dopamine deficiency observed in the Hph-1 mouse negatively affects neural development of amacrine cells (as indicated by fewer TH immunopositive cell processes). In terms of their susceptibility to apoptotic cell death, dopamine has been shown to be pro-apoptotic therefore a reduction in its levels could be protective to the developing amacrine cells^43,44^. Taken together, our findings suggest that a delay in the maturation of retinal interneuron cells, such as amacrine cells lowers theirs susceptibility to oxidative-induced cell death following ischaemic insult.

**Figure 7:**
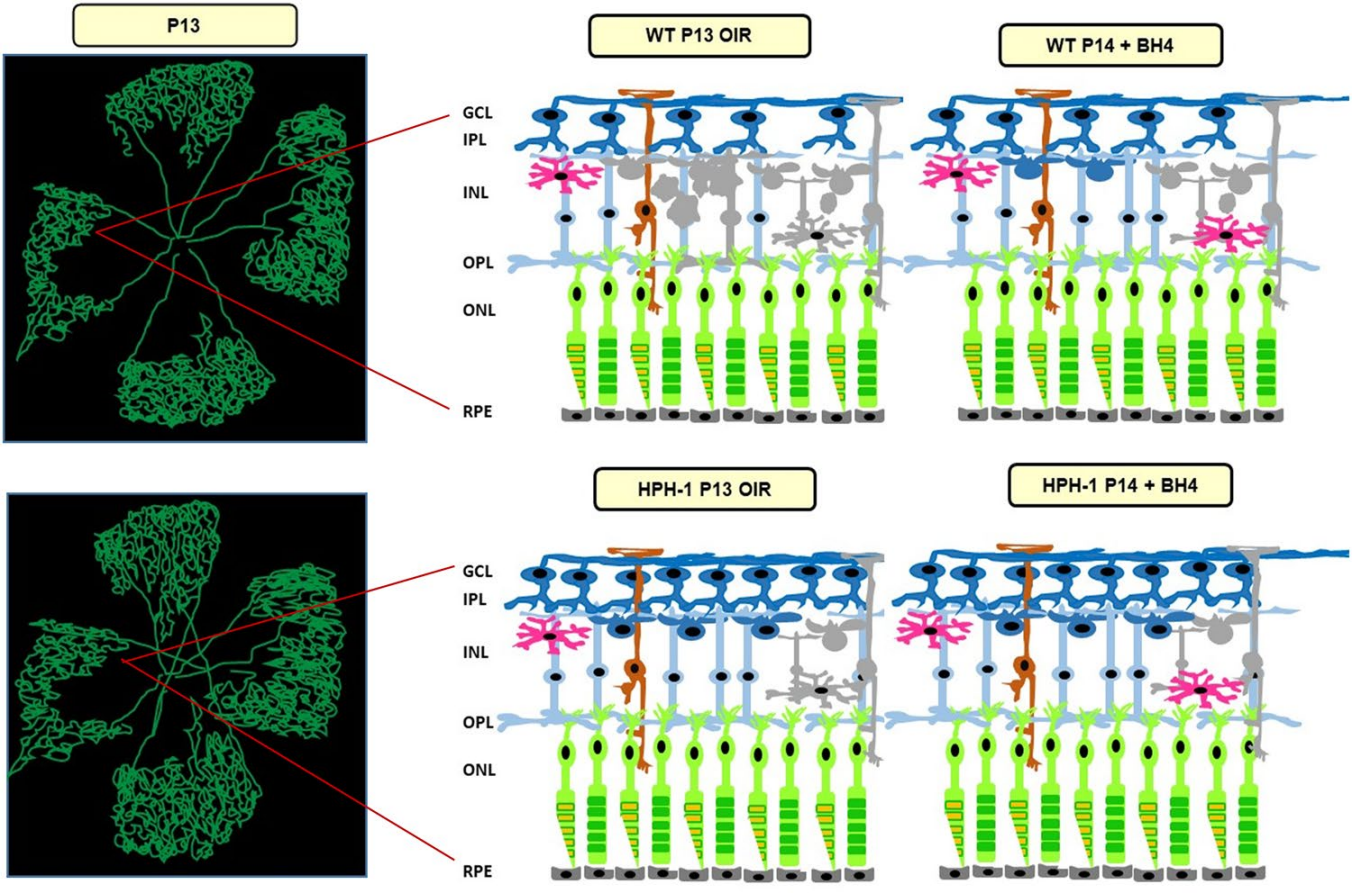
Consequences of retinal ischaemia in Hph-1 and WT animals and the effect of BH_4_ supplementation in lowering apoptotic cell death in the inner retina. Acute ischaemia for 24–48 h (P13–P14 OIR) causes retinal cell death (coloured grey). Hph-1 animals at P13 show evidence of a genotype-related adaption with increased vascular preservation (green in retinal flat mount illustration), lower nitro-oxidative stress and decreased ischaemia-induced apoptotic cell death. Treatment with the stable BH_4_, precursor, sepiapterin, reduces nitro-oxidative stress and cell death in the ischaemic retina. This reduction was most significant in the WT group, in line with their more pronounced responsiveness to hypoxia-induced cell death. Together these findings suggest that reducing nitro-oxidative stress in the inner retina will preserve neural function in response to ischaemic damage.

In addition to changes in neural cell maturation dynamics we also investigated if the Hph-1 genotype showed any differences in the levels of inflammatory cells that may have been responsible for their paradoxical decrease in apoptosis and cell death levels. For example, MG cells have been shown to both induce cell death or act in a neurotrophic manner. Thus next, we determined the extent of MG localisation to the INL. Interestingly, MG were more abundant in the Hph-1 group, again suggesting a developmental increase, possibly related to their higher VEGF levels pre-hypoxia and reported in other studies using this model^33,34^. Notably, they were localised to the same zone as the TUNEL positive cells and contained one or more DAPI positive nuclei in their processes which were clearly separate from the nucleus of the host cell demonstrating engagement in the uptake of neighbouring apoptotic cells. Taken together, these findings suggest that the increased protection against ischaemia and decreased TUNEL positive cells observed in the Hph-1 retinas at P13 is due to a developmental adaption as previously reported and suggested by studies in human disease (Fig. 7 summary image).

Next, after having established the baseline responsiveness of Hph-1 neonates to ischaemic insult we next investigated the impact of BH_4_ on neural damage at P14. With regards to the effect of BH_4_ supplementation at P14, 48 h post-ischaemia comparison of genotypes showed a difference between each of the Hph-1 subgroups. BH_4_ supplementation reduced INL apoptosis in WT but made only a modest improvement in Hph-1 mice which was related to the already low levels of apoptosis in these animals (Fig. 7). Notably the lower levels of apoptosis correlated again to lower NOS activity levels in tissue extracts. Compared to WT mice, Hph-1 littermates showed a similar marked reduction in the level of NOS-related superoxide generation during the ischaemic phase of OIR as shown at P13. Superoxide levels in all three groups were further diminished by BH_4_ supplementation with sepiapterin. This also correlated with a sepiapterin-induced reduction in nitrotyrosine (NT) levels which was more pronounced in WT mice than the Hph-1 littermates. The latter showed no further NT reduction with BH_4_ supplementation, again correlating to their NO production. Taken together this was suggestive of lower NO output and thus lower peroxynitrite levels. Taking the NOS and NT results together our results indicate improved NOS activity. Indeed, improved NOS activity with BH_4_ supplementation has been reported to protect against pathology in ischaemia–reperfusion or reoxygenation injury and to increase blood flow recovery following hindlimb ischaemia^45–47^. Significantly, NT adducts were again highest at the inner aspect of the INL, corresponding to the region of maximal apoptosis. Thus, the reduced apoptosis in the INL following BH_4_ supplementation suggests that BH_4_ has a protective effect during ischaemia. Also, the colocalisation of nitrotyrosine and apoptosis in, and proximal to amacrine cells of the INL, may be related to the fact that the majority of NOS-dependent retinal neurons reside in the INL. In this regard it is significant that the amacrine cells that predominate at the inner aspect of the INL represent the main class of nNOS-dependent neurons in the retina, with some, but less nNOS expression in bipolar cells^1,2,7,48^. Therefore, it is reasonable to propose that local NO production by nNOS expressing neurons represents the source of the INL peroxynitrite, which causes nitro-oxidative damage and cell death in the source cells. Mechanistically, peroxynitrite has been shown to have 2 distinct mechanisms of action in its interaction with NOS: it can either uncouple the enzyme by oxidation of bound BH_4_ or abolish enzymatic function by oxidative destruction of the NOS heme site, thus rendering an inactive enzyme, as opposed to one that is uncoupled^49,50^. In comparison to a range of other oxidant species, including superoxide, hydroxyl radical and hydrogen peroxide, only superoxide and peroxynitrite proved inhibitory to NOS function at concentrations encountered in pathological situations in vivo^8^. Peroxynitrite proved the most destructive, for while BH_4_ was able to fully restore NOS inhibition induced by superoxide, only partial restoration was possible following exposure to peroxynitrite^51^. Therefore, improvement of NOS dysfunction by BH_4_ supplementation with sepiapterin probably involves both reversal of NOS uncoupling by replenishing BH_4_, and protection of the oxidase site by scavenging of peroxynitrite due to the antioxidant properties of BH_4_.

We also examined the expression of inflammatory mediators previously shown to be elevated by hypoxia^7,12,17,22^. Interestingly, BH_4_ supplementation during ischaemia coincided with an increase in the expression of inflammatory cytokines indicating benefits in neural protection rather than the converse. MG are the likely source of these mediators and are normally localised to the plexiform layers of the retina, but may infiltrate the INL during retinal ischaemia^12^. The dual functions of MG have implicated them in inducing cell death and in resolving cell damage. Indeed, our previous findings have shown that INL localised MG are associated with lower ischaemia-induced cell death, therefore we sought to investigate how sepaipterin impacted the expression of inflammatory mediators^7,12,17^. Here we showed that BH_4_ supplementation significantly altered the expression pattern of a range of cytokines measured at P14, and although some anti-inflammatory cytokines were upregulated, the overall profile was pro-inflammatory with major upregulation of TNFα, Il-1, and IFNγ. This finding was in the absence of an effect on MG number suggesting a change in the status of the cells. MG cells have previously been shown to provide neurotrophic support to the brain and retina, and even typical pro-inflammatory cytokines, TNFα, Il-1, and IFNγ, have neurotrophic functions^52–56^. Therefore, taken together with the decreased apoptotic cell death, our findings suggests that the cytokine cocktail in the presence of BH_4_ was neuroprotective, not injurious. Indeed this finding is similar to studies of ischaemic damage in the brain which show MG-mediated protection occurs in the presence of similar cytokines that we show are elevated here in the ischaemic retina, including sICAM, INFγ, IL-1α, KC(CXCL1), MCP-5 (CCL12), MIP2(CXCL2) and TNFα^57^. This suggests that the presence of MG, regardless of cytokine output, plays a crucial role in protecting against ischaemic insult in the retina as it does in the brain. Interestingly, comparisons of protein and expression analysis showed that increases in protein levels did not always correlate to changes in mRNA transcript levels, suggesting a role for hypoxia or the inflammatory milieu in regulating cytokine protein availability. In the current study, MG co-localising with INL cell apoptosis showed evidence of phagocytosis of TUNEL positive nuclear remnants in the ischaemic retina. Interestingly, phagocytic activity in macrophages is associated with induction of an anti-inflammatory phenotype and decreased iNOS-mediated free radical production and NO/RNS/ROS output^58^. However, in other studies, iNOS expression and NO activity is associated with increased phagocytosis and apoptotic cell uptake^59–61^. Thus, it is possible that the effects of MG and macrophage phagocytosis diverge sufficiently to account for these differences. However, more importantly, in the current study, the correlation between MG presence and enhanced cytokine output with lower neural cell death is suggestive of their neurotrophic role in the ischaemic retina. Also, whilst not explored here it is possible that sepiapterin treatment increased the infiltration of immune cells into the ischaemic retina. Thus a component of the inflammatory mediators and cytokines could have originated from these cells. Notably, however, even if this was the case their presence was still associated with lower levels of INL localised apoptosis suggesting a pro-resolving role for these cells.

In summary, here whilst Hph-1 animals showed the expected correlation between BH_4_ and NO, this did not lead to an increase in ischaemia-induced neural cell apoptosis. The associated observation that mice with the Hph-1 genotype had lower amacrine cell arborisation was indicative of delayed neural cell maturation and suggested a reason for their resilience to ischaemia-induced damage. Beyond this finding, this study has provided evidence that NO production by neurons is compromised, with uncoupled nNOS generating superoxide which scavenges NO and leads to the production of peroxynitrite. Together this leads to peroxynitrite-mediated cytotoxicity in NOS-dependent retinal neurons in WT mice that occurs as a result of retinal ischaemia and is rescued by treatment with BH_4_. In parallel, the expression of inflammatory cytokines is suggestive of enhanced MG cell activity that is neurotrophic rather than detrimental, providing further evidence of a pro-resolving role for MG in retinal ischaemia. Together our results demonstrate that BH_4_ supplementation exerts a neuro-protective effect during the ischaemic-hypoxic phase of OIR by modulating the cellular redox state.

## Materials and methods

### GTPCH-deficient Hph-1 (hyperpenylalaninemic) mouse model

GTPCH-deficient Hph-1 mice on a C57/BL6J background were obtained from Professor K Channon (Cardiovascular Medicine, University of Oxford, Oxford, UK) and genotyped as previously described^6,62^. We have previously screened these animals for the presence of the RD8 rhodopsin gene mutation, found in some C57BL6J subvariant strains, and confirmed that they were negative. Breeding pairs of heterozygote animals were routinely used to provide litters of experimental animals containing Hph-1 homozygotes (Hph-1^−/−^), Hph-1 heterozygote (Hph-1^+/−^) animals and wild type (WT) littermate control animals. Comparisons between Hph-1^−/−^ and WT are shown throughout unless otherwise stated. Depending on the assay, comparisons were made between WT and Hph-1 genotypes or two treatment groups for ease of assay or to minimise animal use. In addition, since breeding protocols generated more heterozygotes animals, these genotypes were used preferentially for some assays. In terms of experimental design and in order to limit possible confounding effects due to variability between litters or genetic differences across strains, experiments were designed to include animals from multiple litters, typically three independent litters per group and littermate WT animals used as controls^63^. In addition, another possible confounder in OIR studies is the strong genetic component or inter-strain variability of animals in their susceptibility to developing OIR^64,65^. Thus, here we have used a well validated strain of Hph-1 animals that has been backcrossed onto a C57BL6J background, a strain known to induce a highly reproducible response to OIR. Together, the dual strategy of using litter mate comparisons and C57BL6J back crossed animals increases our confidence that any changes observed are due to differences related to BH_4_ deficiency rather than other strain- or litter-related confounders. Furthermore, multiple use of tissue allowed for a direct comparison of BH_4_ levels in the same animal with retinal phenotype. For consistency, data is shown for WT versus Hph-1^−/−^ unless otherwise stated. All data including the Hph-1^+/-^ heterozygotes is compiled as a table to show genotype–phenotype correlations for all three genotypes when conducted (Table S1: Supplementary Data). Please note that the term Hph-1 refers to either the homozygous or heterozygous groups.

### Induction of retinal ischaemia and oxygen induced retinopathy model (OIR)

Ischaemia induction was based on the protocol for the OIR model of Smith et al.^66^. Each independent OIR experiment routinely involved six to seven time-mated breeding pairs. This ensured that litters were born and exposed to oxygen within the same 24 h time frame. Neonatal mice were kept at room air from birth to postnatal day 7 (P7), allowing the development of the normal primary vascular plexus in the retina, which is complete by P7. The mice were exposed to a standard 12-h light–dark cycle. On postnatal day 7 (P7) pups along with their nursing dams were placed into a chamber were the oxygen was maintained at 75% by an Oxycycler (A84X0V; Biospherix, Canada) to induce vascular regression of the central retinal vessels. At P12 the mice were returned to room air resulting in significant hypoxia in the central avascular portion of the retina. Animals were collected at P13 or P14 to determine ischaemia-induced cell damage. Eyes were either fixed in 4% paraformaldehyde (PFA) for 4 h upon collection for retinal flat mount preparation, or embedded and frozen in OCT compound (Sakura Fintek, Zoeterwoude, The Netherlands) for preparation of 10 µm fresh-frozen sections to be used for TUNEL assay, DHE fluorescence staining and NT immunostaining. Alternatively the retinas, lungs or brains were snap frozen in liquid nitrogen and stored at − 80 °C for determination of NOS activity, qPCR analysis, cytokine profiler array or BH_4_.

### BH_4_ supplementation

Sepiapterin (Sigma-Aldrich) was dissolved in sterile dimethyl sulfoxide (DMSO, Sigma). This solution was diluted with sterile saline (sodium chloride 0.9% W/V—Fanin). For the vehicle control (VC), an equal volume of DMSO was diluted in sterile saline solution (final conc.-3.5% [v/v]). At P12 hyperoxia-exposed mouse pups were injected intraperitoneally with the VC or 10 mg/kg sepiapterin. This concentration is well tolerated and results in elevated BH_4_ tissue levels^6,10,67^. Mice were treated with sepiapterin at P12 and the tissue collected at P14.

### Determination of BH_4_ biopterin by high performance liquid chromatography (HPLC) analysis

Tissue BH_4_ level was measured in tissue samples by high performance liquid chromatography (HPLC) as described previously with fluorescence detection after iodine oxidation in acidic or alkaline conditions^6,34^. Previously we showed a correlation between retinal and brain BH_4_ levels in neonatal animals therefore here to maximise the amount of assays conducted we used lung and brain tissue to confirm the effectiveness of sepiapterin supplementation to increase BH_4_ levels^6^. Further confirmation that this finding is consistent across different strains is also shown for C57Bl6 WT animals (Fig. S4) and shows a correlation between BH_4_ measurements in retinal, brain and lung tissue following supplementation with sepiapterin. Tissue samples were homogenized in ice cold extraction buffer (50 mM Tris–HCl pH 7.4, 1 mM dithiothreitol (DTT), 1 mM ethylenediaminetetraacetic acid (EDTA)) and centrifuged at 14,000 rpm for 15 min at 4 °C. To determine total biopterin (BH_4_, dihydropterin (BH_2_), and oxidized biopterin) by acid oxidation, each sample was split into two Eppendorfs with 10 µl acid (equal volumes of 1.5 M HClO_4_ and 2 M H_3_PO_4_) added to one and 10 µl alkali (10 mmol/l NaOH) added to the other. 1% iodine in 2% potassium iodine (KI) solution was then added to both the acidic and alkaline samples, vortexed and incubated in the dark for 1 h at room temperature. Following the 1 h incubation, 20 µl 1 M phosphoric acid (H_3_PO_4_) was added to the basic samples only. Iodine was reduced by adding 5 µl of freshly prepared ascorbic acid solution (20 mg/ml). All samples were then centrifuged at 13,000 rpm for 5 min and the supernatant collected for injection. Samples of 50 µl were injected into a 250-mm-long, 4.6-mm-inner diameter C-18 stationary phase column (5-µm particle size; Phenomenex, Cheshire, UK) eluted with a methanol-15 mM potassium phosphate buffer (8:92, vol/vol) mobile phase running at a flow rate of 0.5 ml/min. Fluorescence detection (350 nm excitation, 450 nm emission) was performed using a fluorescence detector (RF20AXS, Shimadzu). BH_4_ levels were measured by calculating the difference between the peak given by the sample subjected to acid oxidation which determines total biopterin (BH_2,_ BH_4_ and oxidised biopterin) and the peak given by the sample subjected to alkali oxidation which determines BH_2_ levels. BH_4_ concentrations were calculated in relation to the BH_4_ standards and expressed as nanograms per milligram of protein.

### NOS activity assay

NOS activity was determined by quantifying the conversion of Carbon-14 radiolabeled arginine to citrulline using a modified NOS Activity Assay kit (Cayman, Ann Arbor, MI) as described previously^12,34^. Briefly, pooled mouse retinas were homogenized and added to a reaction mixture containing [^14^C] arginine (0.05 µCi; (Amersham Biosciences, Piscataway, NJ) but without additional BH_4_ and incubated for 1 h at 37 °C. This reaction consumes NADPH and also requires molecular oxygen, calcium, calmodulin, and BH_4_. Although BH_4_ is normally used in the assay, additional BH_4_ was not added to the extraction buffer or assay, to ensure that the reaction was driven by endogenous BH_4_ in the sample. Arginase present in samples competes with NOS for arginine so samples were also treated with buffer containing 5 mmol/l of the arginase inhibitor, (S)-(2-boronoethyl)-l-cysteine-HCl (BEC; Calbiochem/EMD Chemicals, Gibbstown, NJ) to ensure only NOS activity was measured. Following the 1-h incubation, unreacted arginine was removed by adding ice-cold HEPES buffer containing EDTA (pH 5.5) and filtration through a spin column. [^14^C]-l-citrulline levels were determined by subtracting background values from the total counts. For each sample, background was determined by incubation in the presence of L-NNA and subtracted from the total counts.

### Cryosections and preparation of slides for TUNEL assay and detection of reactive oxygen and nitrogen species

WT and Hph-1 eyes were embedded fresh in OCT compound in aluminium foil moulds and orientated so that the cornea was facing the side of the mould. To enable fast freezing of the tissue and help prevent the formation of ice crystals, the mould was lowered into liquid nitrogen cooled isopentane (Sigma) using forceps. OCT blocks containing eyes were stored at − 80 °C until sectioning. Prior to sectioning, OCT blocks were left to equilibrate to − 20 °C in a Leica 1900 cryostat (Lecia Biosystems). Eyes were cut into 10 µm sections and collected on Superfrost slides (Thermo Scientific). The slides were left to dry for a minimum of 30 min before being stored at − 20 °C or used for staining.

### Detection of apoptotic cells via TUNEL (TdT-mediated dUTP-nick-end labelling) assay

TUNEL staining was preformed using an in situ cell death detection kit (Sigma-Aldrich—previously Roche UK) and conducted as described previously^12^. Briefly, freshly cut sections were fixed with 3% PFA in PBS for 20 min then washed with PBS for 30 min. They were then permeabilised on ice for 2 min in freshly prepared 0.1% sodium citrate and 0.1% Triton-X-100 solution, and then washed with PBS. As a positive control for TUNEL staining, additional retinal sections were incubated with 5% DNase 1 (Invitrogen) in PBS for 10 min at room temperature followed by five 3 min washes with PBS. The TUNEL reaction mixture (containing the labelling solution and the enzyme) was added to the sections for 1 h at 37 °C in the dark (labelling solution, without terminal transferase, was added to one slide as a negative control). After washing with PBS, Vectashield (Vector Laboratories, H-1200) containing DAPI was added to stain nuclei and prevent photobleaching. Retinal specimens were imaged using a confocal laser microscope (Eclipse TE2000-U) and apoptotic cells identified by fluorescent staining. Images were corrected for background fluorescence using Image J software. The total fluorescence measurement of three images from each section was combined to give a fluorescence value for the section and the mean of eight sections per eye combined to give a mean value for each specimen.

### Detection of reactive oxygen and nitrogen species production

Dihydroethidium (DHE; Life technologies) was used to measure reactive oxygen species production in-situ in fresh tissue samples in the presence and absence of inhibitors which are added to the tissue samples ex vivo^12^. This assay measures the presence and activity of ROS producing enzymes at the time of tissue collection. Sections directly adjacent to those used for the TUNEL staining and from the same ischaemic region where used. A Dako hydrophobic pen was used to draw a ring around the sections before incubating with the NOS inhibitor *N*_ω_-Nitro-l-arginine methyl ester hydrochloride (1 mM L-NAME) (Sigma) or superoxide dismutase–polyethylene glycol (PEGSOD; Sigma) (400 U/ml) in PBS for 30 min at room temperature (RT). Then DHE (10 µM in PBS) in the presence and absence of the same inhibitors was added and sections incubated for 1 h at 37 °C in a dark, humidified chamber. Slides were then washed with PBS and imaged using a confocal laser microscope with the same image acquisition settings and exposure time maintained throughout collection of all the images, thus allowing fluorescent intensities to be compared. The inner nuclear layer (INL), where the TUNEL positivity had been most prominent, was demarcated and the fluorescence intensity quantified using ImageJ software. The average fluorescence value was determined from eight non-overlapping images per eye.

### Nitrotyrosine adduction

Peroxynitrite and tyrosine residues in proteins react to form nitrotyrosine (NT), thus NT protein adduction is widely used as a surrogate marker of peroxynitrite formation^68^. Briefly, frozen retinal sections were fixed for 30 min in 4% PFA, then rinsed with PBS before being permeabilized with 0.2% Triton X-100 in PBS. Sections were blocked for 30 min with 5% goat serum in PBS with 0.1% Triton-X-100 and 1% BSA. Anti-nitrotyrosine antibody (Upstate) diluted 1:200 in 2% goat serum was then applied overnight at 4 °C. Sections were washed three times with PBS containing 0.1% TritonX-100, then incubated with secondary antibody, Alexa Fluor goat anti-rabbit 568 (Life Technologies, Paisley, UK) diluted 1:500 for 1 h. After further washing, sections were incubated with DAPI for 20 min before being mounted with Vectashield mounting medium (H-1000). Using Image J software, the INL was demarcated and the average fluorescence value was determined from six sections and three eyes from three independent litters.

### Tissue preparation and immuno-staining retinal flat-mounts with TH and quantification of cell bodies

One eye from each animal was fixed in 4% PFA for 4 h followed by one wash in PBS and eyes prepared for flat mount preparation as described for lectin staining. The eyes were permeabilised/blocked overnight at 4 °C on a rocking platform in PBS containing 1% goat serum, 0.5% Triton X-100 and 0.1 mM CaCl2 (permeabilisation buffer). The following day eyes were incubated in tyrosine hydroxylase antibody (Millipore) (1:200) in permeabilisation buffer for 4 h at 37 °C in a humid chamber. Eight washes (15 min each with continuous shaking) were carried out in the permeabilisation buffer at 37 °C. Goat anti-rabbit secondary antibody (1:500) (Invitrogen) was incubated for 3 h at 37 °C before washing 8 times in permeabilisation buffer. To visualise the retinal vasculature eyes were incubated with isolectin B4 as described below. Finally, retinal flat-mounts were washed and mounted in Vectasheild. Images of the immunostained retinal flat-mounts were acquired using a Leica DMi8 fluorescent microscope and the Leica Application Suite X programme was used to stitch flat-mount images together which enabled visualisation of the whole retinal flat-mount (Figs. S2, S3). TH positive cell bodies were counted in the entire retina of P7 from all genotypes and no significant difference in number of cell bodies was found. Three P7 retinas from 3 independent litters were analysed per genotype were analysed.

### Quantification of TH immuno-staining in P7 mouse retina

TH immunopositive processes were quantified from high power z-stack images taken on a confocal laser microscope (Eclipse TE2000-U) using the × 40 objective lens. For consistency, similar locations of each retina were captured and all images for quantification were captured from an arteriole in the mid-peripheral area of the retina (Fig. S2). Arterioles were described as vessels with numerous capillary free zones directly adjacent to the vessel. Confocal z-stacks (15 × 2 µm) were taken through the entire thickness of the TH-immunopositive network which included the IPL and INL. The average length of the TH positive processes was assessed using the grid cross point system^69^. A grid was applied to each image dividing it into 9 × 9 equal sized squares. Three retinas from three independent litters were analysed per experimental group and four images were taken per retina (1 per quadrant). Processes were quantified by the number of squares that they crossed (Figs. S2, S3) and expressed as the total number of squares covered.

### Cytokine profiler array

The mouse cytokine array panel A (R&D systems) was used to determine the effect of sepiapterin treatment on the expression of a range of cytokines. Firstly, the nitrocellulose membrane strips containing the arrayed antibodies were incubated in array buffer for 1 h on a rocking platform. Retinal samples were prepared by homogenising in PBS containing protease inhibitors (Roche) and Triton X-100 added to yield a final concentration of 1% V/V to each sample. Samples were frozen and thawed twice to aid dissociation of the cell membranes. Samples were then diluted, mixed with a cocktail of biotinylated detection antibodies and the mixture applied to the membrane strips containing the cognate immobilised antibodies spotted in duplicate. Any cytokine-antibody complex present in the sample then binds to these spots. Following overnight incubation at 4 °C, membranes were washed with wash buffer before incubation with Streptavidin-HRP for 30 min at RT. Membranes were washed for a further three times for 10 min each and chemiluminescent detection reagent added. Membranes were covered and placed in an autoradiography film cassette and exposed to X-ray film. The resulting film was scanned at 600DPI and the average pixel density determined using Image J. To determine the relative change in cytokine levels across treatment groups the average value for the two replicate spots was calculated and the value of the negative control spots subtracted.

### Real-time polymerase chain reaction (RT-PCR)

RNA was extracted using the miRNeasy Mini Kit according to the manufacturer’s instructions (Qiagen). Retinas were lysed directly in QIAzol lysis reagent (50 µl) and homogenised for 20 s. A further 150 µl QIAzol was added and the lysate left at room temperature for 5 min. Chloroform (Sigma) was added to the lysate, the samples shaken vigorously before centrifugation at 12,000 rpm, 4 °C for 15 min. The RNA containing upper phase was transferred to a new tube, ethanol added and the mixture transferred onto an RNeasy Mini spin column. On-column DNase digestion was carried out on the samples with an RNase-Free DNase kit (Qiagen) to ensure no DNA was present in the sample. DNase was efficiently removed in subsequent wash steps. RNA was eluted from the column with water. The integrity of the RNA was confirmed by the presence of clearly visible 28s and 18s ribosomal RNA bands following electrophoresis on a 1% agarose gel. The Superscript VILO cDNA Synthesis Kit (Thermo Fisher Scientific) was used to generate cDNA for use in RT-PCR. Equivalent amounts of RNA (500 ng) was used in each reaction and 5X VILO reaction mix and10× SuperScript enzyme mix added and samples reacted at 42 °C for 1 h. The reaction was stopped by a 5 min incubation at 85 °C before being cooled to 4 °C. In negative control samples SuperScript was omitted to prevent the reverse transcription reaction occurring confirming the absence of DNA. Following cDNA synthesis the samples were diluted 1:5 with nuclease free H_2_O for use in the PCR reaction using LightCycler® 480 SYBR Green I Master mix (Roche) and forward and reverse primers added. Primers were designed in house using Primer-BLAST (Basic Local Alignment Search Tool) and synthesised by IDT (*Gch1*, *Hif1α*, *iNOS*, *Ccl2*, *Il1α*, *Il1β*, *Arg1*, *Arg2* and *Mip2*) or purchased pre-designed from Sigma (*Vegfa*, *eNOS* and *18S*). All primers were used at a working concentration of 2.5 µM. 18S ribosomal RNA was used as an appropriate housekeeping gene in the OIR model as it was not subject to change with fluctuating oxygen levels^12^. The average quantitation cycle (Cq) value was calculated and the mean Cq value for the housekeeping gene subtracted to determine ΔCq. Experimental samples were then normalised to control samples to determine ΔΔCq values. The data was quantified using the formula: 2^−∆∆Ct^. The specificity of the reaction was confirmed by melting-curve analysis.

### Retinal flat mount preparation, staining and analysis

*B. simplicifolia* lectin (GS isolectin B4) was used to visualise vascular coverage in flat mounts from P13 animals as described previously^34^. Briefly, mouse eyes were fixed in 4% PFA for 4 h at room temperature and washed with PBS before dissection. Four incisions from the retinal periphery to the optic nerve were then made to allow the eye cup to be flat-mounted. Once dissected the eyes were incubated in permeabilisation/blocking buffer—PBS with 0.5% Triton-X-100, 1% goat serum, 0.1 mM CaCl_2_, for 4 h at 37 °C or overnight at 4 °C. The next day specimens were washed with permeabilisation buffer eight times at 37 °C. Specimens were incubated with GS isolectin B_4_ (Sigma L2140) (20 µg/ml) in permeabilisation buffer for 4 h at 37 °C. Eye cups were then incubated with Alexa-Fluor-488 or 568 streptavidin conjugate (Molecular Probes, S11226) diluted 1:500 in permeabilisation buffer. This was applied for 3 h at 37 °C in the dark before being washed a further eight times with permeabilisation buffer. The specimens were incubated with the nuclear stain DAPI at 1:1000 in PBS (Molecular Probes, D1306) for 1 h at 37 °C. Following this, the eye cups were washed with PBS containing 0.1 mM CaCl_2_. Eye cups were flat-mounted in Vectashield (Vector Laboratories, H-1000) onto slides and coverslips sealed on top. The slides were viewed using a Leica DMi8 Microscope or a confocal microscope. Following staining low power images (× 5 objective) of the whole specimen were captured and automatically stitched together. NIS elements analysis software was used to demarcate the vascular (lectin stained vessels with a typical chicken-wire morphology) and avascular (no lectin staining) areas of the retinal flat-mounts. Vascular and avascular areas were quantified as a percentage of the total area of each retina.

### Quantification of microglial cell density

Microglial (MG) cell density was estimated from GS isolectin B4 stained flat mounts from P13 and P14 animals and confocal microscopy imaging as described previously^12,35,36^. MG cells, characterized by their typical dendritic morphology, have many processes that span a number of layers, therefore for quantification a z-stack of 2 µm images were recorded from the full thickness of the inner retina in the ischaemic regions of the central retina using a × 40 objective. One z-stack image was taken from each quadrant and the average of 4 were used to calculate a mean value for each retina. The position of these cells in the INL was confirmed in relation to the nuclear staining of the inner and outer nuclear layers which form a very typical striated pattern in z-stack confocal images^12^.

### Statistics

As noted above, in order to limit effects due to variability between litters experiments were designed to include sample animals from multiple litters, typically three independent litters per group unless stated otherwise^63^. Three neonatal eyes from three different OIR litters were analysed per experimental group for, TUNEL assays, and DHE/NT fluorescence. For NOS activity assays, three or four retinas were pooled for each group from different OIR litters and assayed in triplicate. For vascular area three to seven retinas were analysed per group. Two retinas from separate animals were used per cytokine profiler array (one representative experiment is shown). For RT-PCR one retina was used from three independent litters and the results combined post-processing. For the MG quantification, 7–13 flat mounts were used. RT-PCR experiments from the P7 retinas was carried out using 3 separate litters (Fig. S3). An independent Student’s t-test was used to determine the statistical significance between two groups. ANOVA was used to determine statistical significance between three or more groups followed by a Bonferroni post hoc test. Statistical significance was defined as *P < 0.05; **P < 0.01 and ***P < 0.001. Data is presented as average ± standard error of the mean (SEM). Data containing a description of all genotypes is included in Table S1: Supplementary data.

### Ethical approval

All procedures were conducted in accordance with ARRIVE guidelines, the Association for Research in Vision and Ophthalmology (ARVO) statement for the use of animals in ophthalmic and vision research and the Animals Scientific Procedures Act (ASPA) 1986 with prior approval from the Queen’s University Belfast Animal Welfare Ethical Review Body (AWERB).

### Supplementary Information

Supplementary Information.

## Data Availability

The data used to conduct this study are available from the corresponding author upon reasonable request.
