# Supplementary material for: BH4 supplementation reduces retinal cell death in ischaemic retinopathy
Source: Sci Rep. 2023 Dec 2;13:21292. doi: 10.1038/s41598-023-48167-5 (PMC10693630; doi:10.1038/s41598-023-48167-5)
Supplement: Supplementary file 1 — Supplementary Information. [file 41598_2023_48167_MOESM1_ESM.docx]

**Supplementary Information for**

**BH4 supplementation reduces retinal cell death in ischaemic retinopathy**

*Kevin S Edgar, Ciara Cunning, Tom A Gardiner, Denise M McDonald* *

Wellcome-Wolfson Institute for Experimental Medicine, Queen’s University Belfast, 97 Lisburn Road, Belfast BT9 7BL, UK.

*Corresponding author:

Denise M McDonald

**Email:**  [d.mcdonald@qub.ac.uk](mailto:d.mcdonald@qub.ac.uk)

**This file includes:**

Supplementary Figure S1

Supplementary Figure S2

Supplementary Figure S3

Supplementary Figure S4

Supplementary Figure S5

Supplementary Figure S6

Supplementary Figure S7

Supplementary Table S1

**Supplementary Data**

**Figure S1 Nitrotryosine levels are reduced in P13 OIR Hph-1**^-/-^ **mice compared to WT controls** Nitrotyrosine (NT) immunoreactivity was used as an indicator of peroxynitrite formation. NT fluorescence intensity in the inner nuclear layer (outlined area shown in Figure 3D) was measured in retinal sections from mice following OIR at P13. (A) and (B) show two independent experiments out of three (shown in Figure 3 D-E) and shows a consistent reduction in Hph-1^-/-^ mice across experiments ****p<0.001. For each animal 6 retinal sections were analysed and three images were taken per section.

**Figure S2 Quantification of TH immunopositive processes in P7 mouse retinas.** (A) Using the vasculature as a reference point, confocal z-stacks (which did not include the superficial vasculature) were taken below an arteriole from the mid-periphery of the flat-mount as outlined by the yellow squares in panel A. (B) TH positive cell bodies and processes (red) were located at the junction between the INL and IPL. Note the long processes extending from the cell body. (C) and (D) Higher power image demonstrating how the grid crosspoint system was used to analyse the TH positive processes in the P7 mouse retina.

**Figure S3 TH analysis shows a developmental reduction in the spread of amacrine cell dendrites in Hph-1^-/-^ retinas compared to WT controls which is associated with dysregulated expression of differentiation markers.**  (A) P7 mice displayed a significant decrease in the coverage of TH positive processes in Hph-1^-/-^ mice compared to WT. The lower panel shows pericellular rings of adult animal retinas. Retinal sections (right hand panel) were immunostained with TH antibody and the nuclear stain DAPI to ease visualisation of the retinal layers. The right hand panel shows the location of amacrine cells in the inner nuclear layer (INL) of a P7 retinal section. The yellow boxed area (lower right) highlights a single TH positive cell with TH positive processes extending from the cell body. Upper right hand panel shows the yellow boxed area at higher magnification. (B) Quantification of TH immunopositivity in P7 retinal sections shows that the total TH coverage was significantly lower in Hph-1 animals. By adulthood the TH positive processes had developed to form a network of pericellular rings (lower left hand panel in A). Quantification showed a small but not statically significant decrease in the number of TH positive pericellular rings in adult Hph-1 mice compared to WT. For quantification, four images were taken per retina (1 from each quadrant) and three P7 retinas were analysed per experimental group from 3 independent litters. (C) Gene expression in P7 WT and Hph-1^-/-^ mice. Four P7 retinas from 2 littermate mice were pooled per genotype. RT-PCR experiments were carried out from 3 separate litters (n=3). Genes were normalised to the housekeeping gene 18S. *GCH1* as expected decreased from WT to Hph-1^-/-^ as well as *TH*. Expression of the cell fate markers *Notch4* and *Sox17* were also significantly decreased in Hph-1^-/-^ (* p< 0.05; **p<0.01; comparison to the WT).

**Figure S4 Correlation between BH_4_ levels in lung, brain and retinal tissues following BH_4_ supplementation in C57Bl6 animals**: **Intraperitoneal supplementation of sepiapterin effectively increases BH_4_ level across multiple tissues.** Mice supplemented with sepiapterin at P7 before tissue collection at P9 showed a significant increase in tissue BH_4_ level in **(**A) retina, (B) brain and (C) lung. HPLC analysis confirmed an increase in BH_4_ levels in all tissue following BH_4_ supplementation. Comparison of dihydropterin (BH_2_) and BH_4_/BH_2_ levels following BH_4_ supplementation. Dihydropterin (BH_2_) levels were unchanged following sepaipterin supplementation in retina (A), lung (B) and brain (C) samples. This resulted in BH_4_/BH_2_ ratios that indicated preservation of BH_4_.  For direct comparison, retina, brain and lung from the same animal was processed; retina n=3 independent experiments with n=5 retinas assayed in duplicate per experiment; n=2 independent experiments for brain and lung using n=2 samples assayed in duplicate per experiment. *p<0.05; ** p< 0.01.

**Figure S5 Sepiapterin elevated BH_4_ levels in brain and lung 48h post-injection: Comparison of BH_4_ levels in tissues from WT and Hph-1^-/-^ animals at P14 following BH_4_ supplementation**. (A) HPLC analysis confirmed the incremental decrease in BH_4_ levels in the lungs of Hph-1^-/-^ animals compared to WT. An increase in BH_4_ levels in all genotypes was confirmed following BH_4_ supplementation with WT and Hph-1^+/-^ being significant. (B) HPLC analysis of the brain also confirmed an incremental decrease in BH_4_ levels across the genotypes. Sepiapterin supplementation also increased BH_4_ levels in all groups. (C-D) Comparison of dihydropterin (BH_2_) and (E-F) BH_4_/BH_2_ levels in tissues from WT and Hph-1^-/-^ animals at P14 following BH_4_ supplementation. Dihydropterin (BH_2_) (C-D) levels showed a similar stepwise decrease in levels across genotypes and increase following sepaipterin as shown for BH_4_ in lung (A) and brain (B) samples. This resulted in BH_4_/BH_2_ ratios that indicated preservation of BH_4_ (E-F). Brain and lung from the same animal was processed; n=2 independent experiments using n=2 tissue samples from independent animals assayed in duplicate. *p<0.05; ** p< 0.01.

**Figure S6 Sepiapterin supplementation increased the levels of inflammatory markers without evidence of a measurable change in INL localised MG cell numbers in P14 Hph-1^+/-^ heterozygote animals.** (A-B) The number of microglial cells (MG) in retinal lectin-stained flat mounted retinas were quantified. (B) Microglial cell density was estimated from *B simplicifolia* lectin (GS isolectin B4) stained flat mounts and a series of z-stack images taken throughout the entire retina 2µm apart were taken in the ischemic region of the central retina for quantification. (B) There was no significant difference in the numbers of MG cells between treatments (n=7-13 group). Scale bars are 100µm. n≥ 7 per group from 6 litters (C-D) Cytokine profiler analysis of P14 OIR retinal samples of mice treated with sepiapterin showing increased pro-inflammatory cytokine levels. Retinas were homogenised and applied to membranes containing cytokine capture antibodies arrayed in duplicate. Membranes were then placed in chemiluminescent detection reagent and exposed to X-ray film. Corresponding signals were compared between the sepiapterin treated and untreated (VC treated) membranes to determine the change in cytokine levels. ICAM ^(1)^, KC/CXCL1 ^(2)^, TIMP-1 ^(3)^ and TNF-α ^(4)^ showed the most pronounced increases following BH_4_ supplementation. n=2 retinas from independent animals per treatment; **p<0.01; *** p< 0.001 comparison to VC.

**Figure S7 Expression of cytokines and inflammatory markers in retinal samples from VC and Sepiapterin treated mice.** Retinas of P14 OIR heterozygote Hph-1 mice injected with sepiapterin or VC were analysed by RT-PCR for select inflammatory related gene targets and showed a trend toward increased retinal expression following sepiapterin supplementation. Fold change in gene expression following sepiapterin supplementation is shown; n= 3 retinas from 3 independent litters.

**
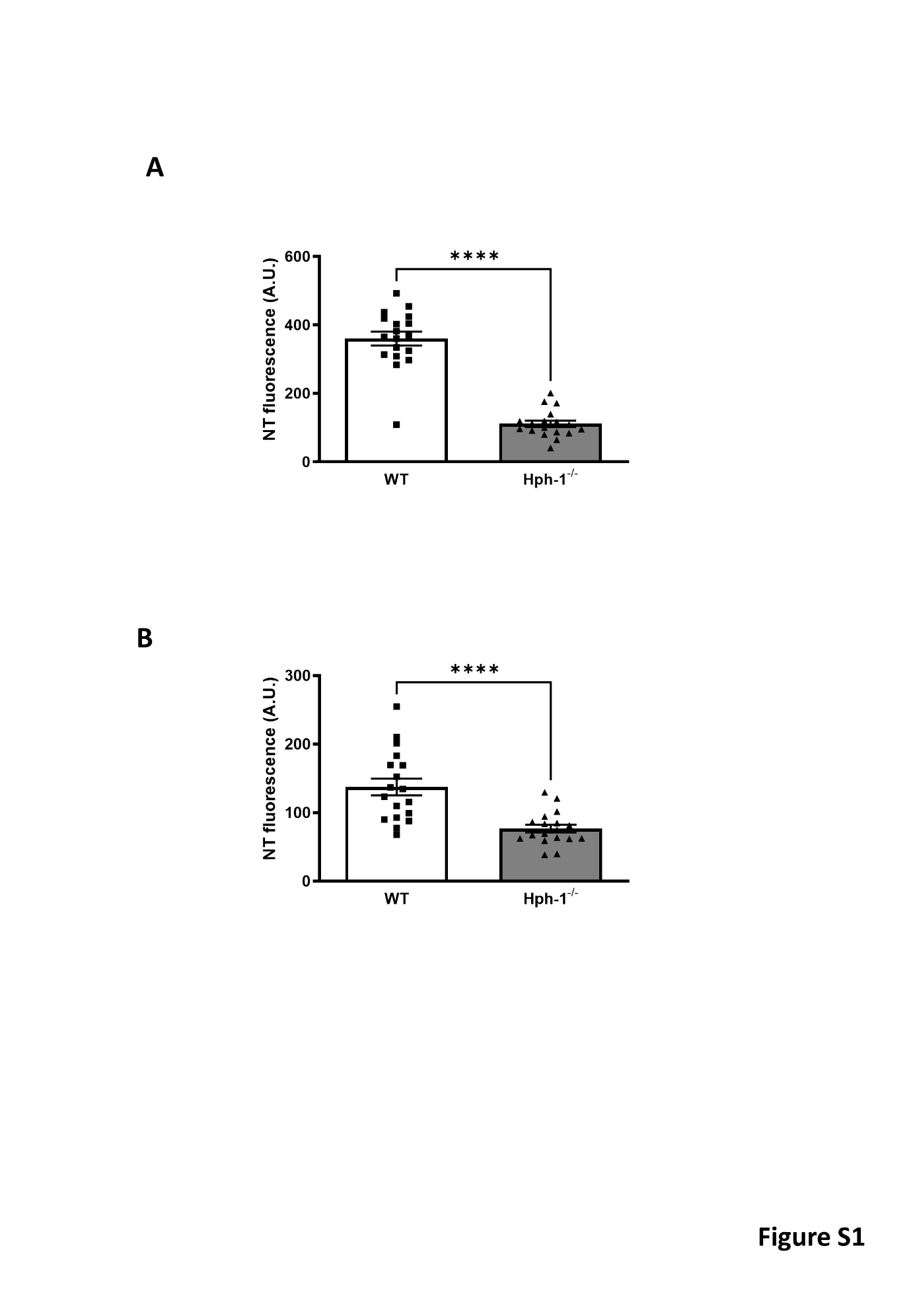
**

**
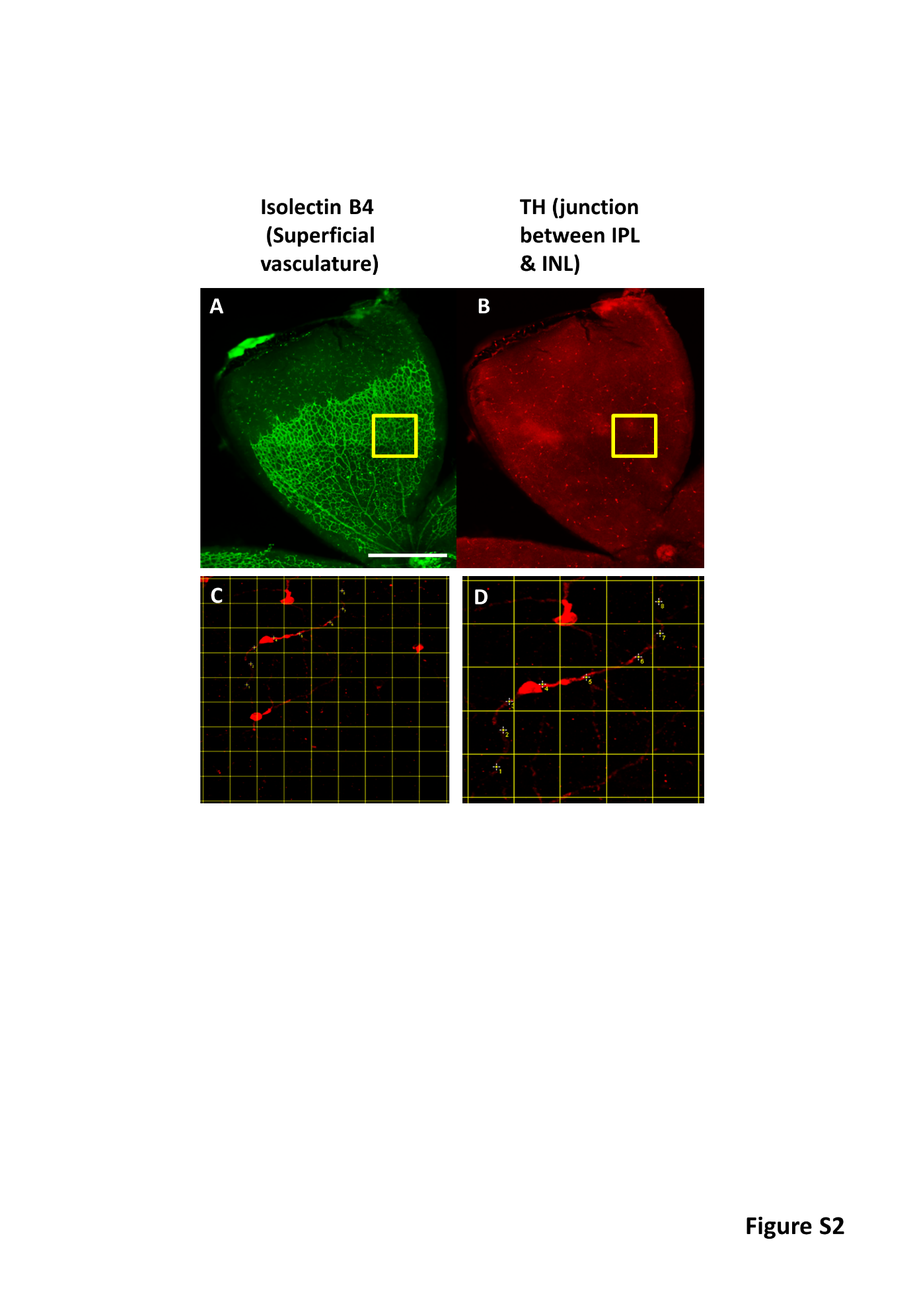
**

**
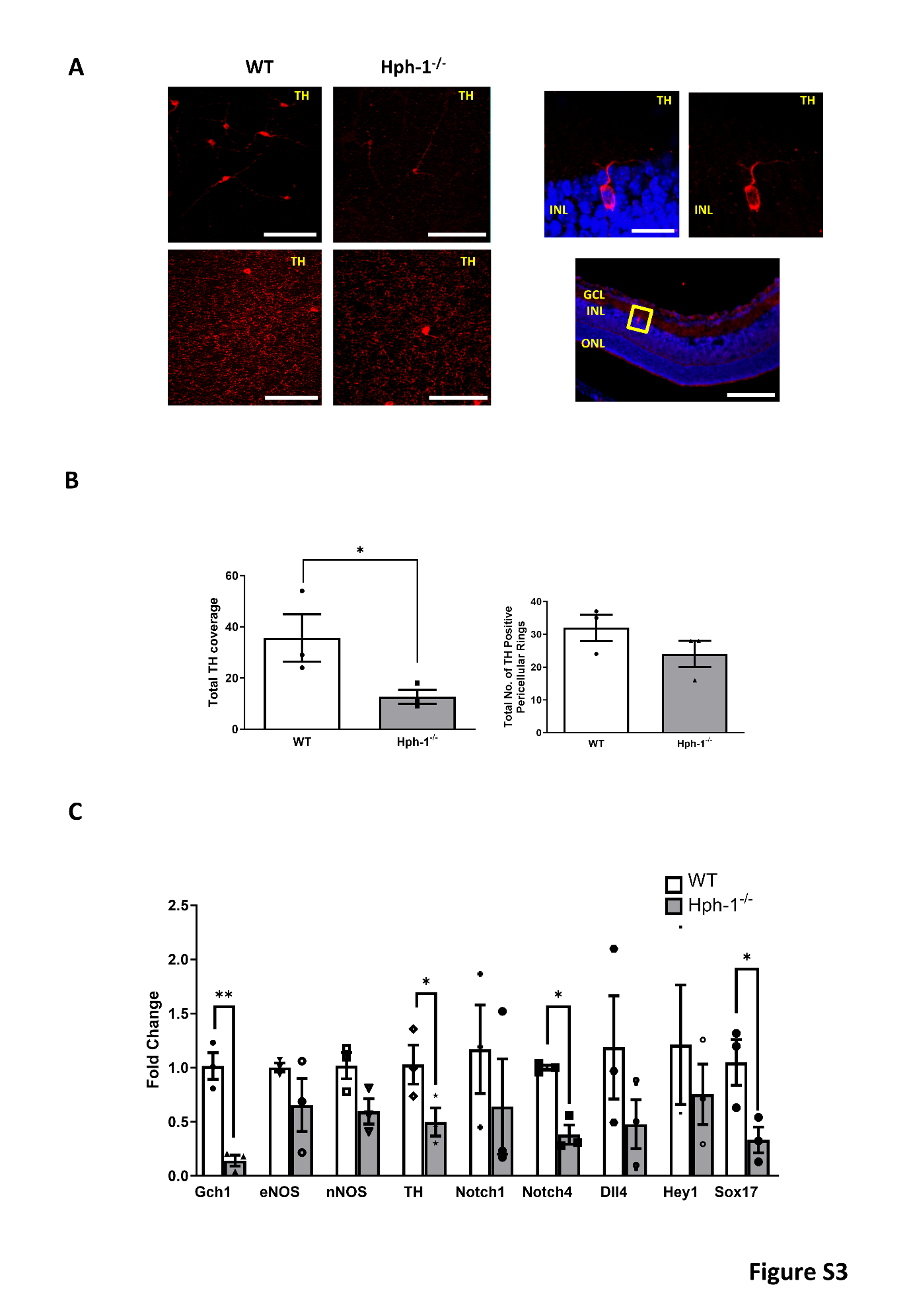
**

**
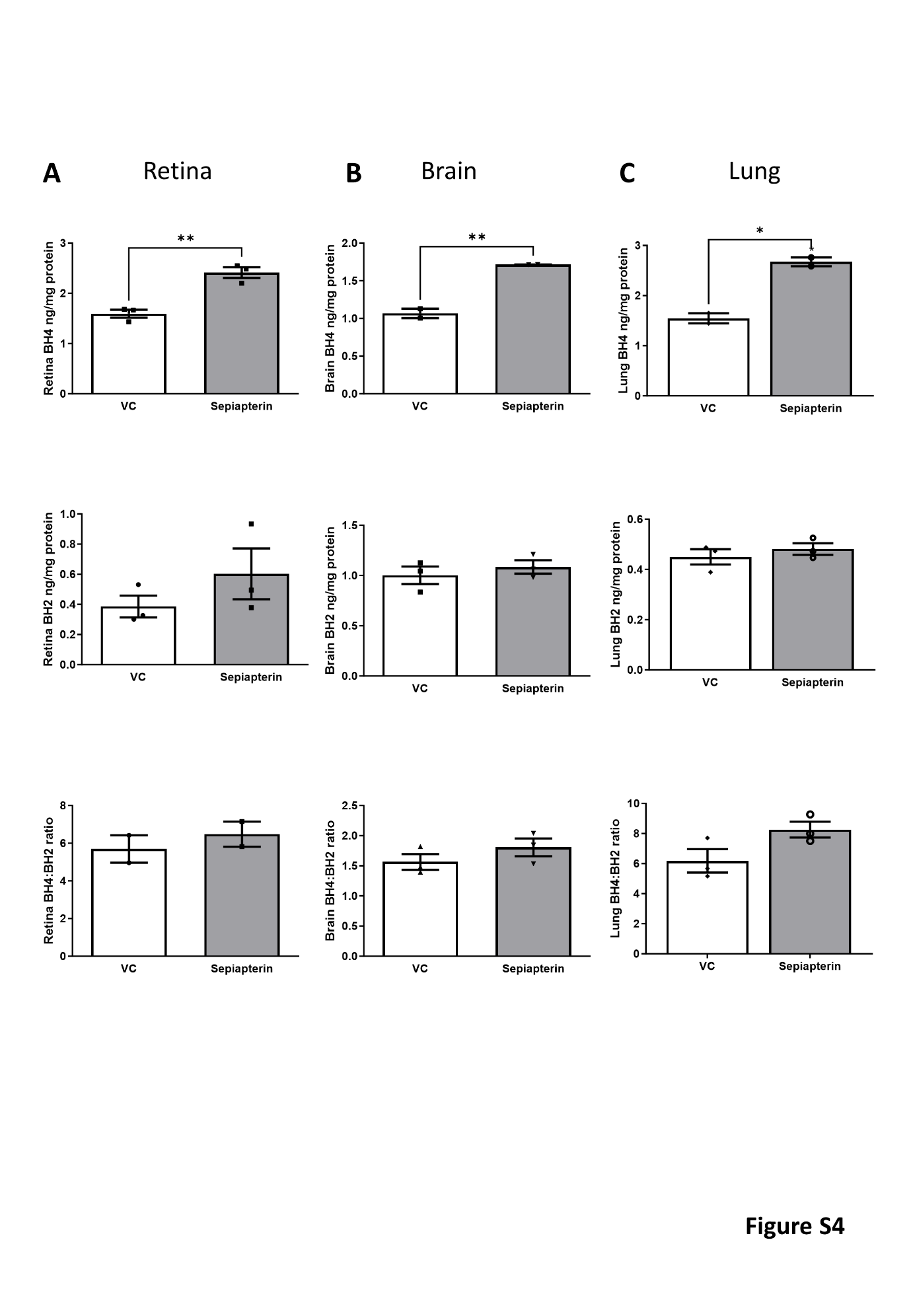
**

**
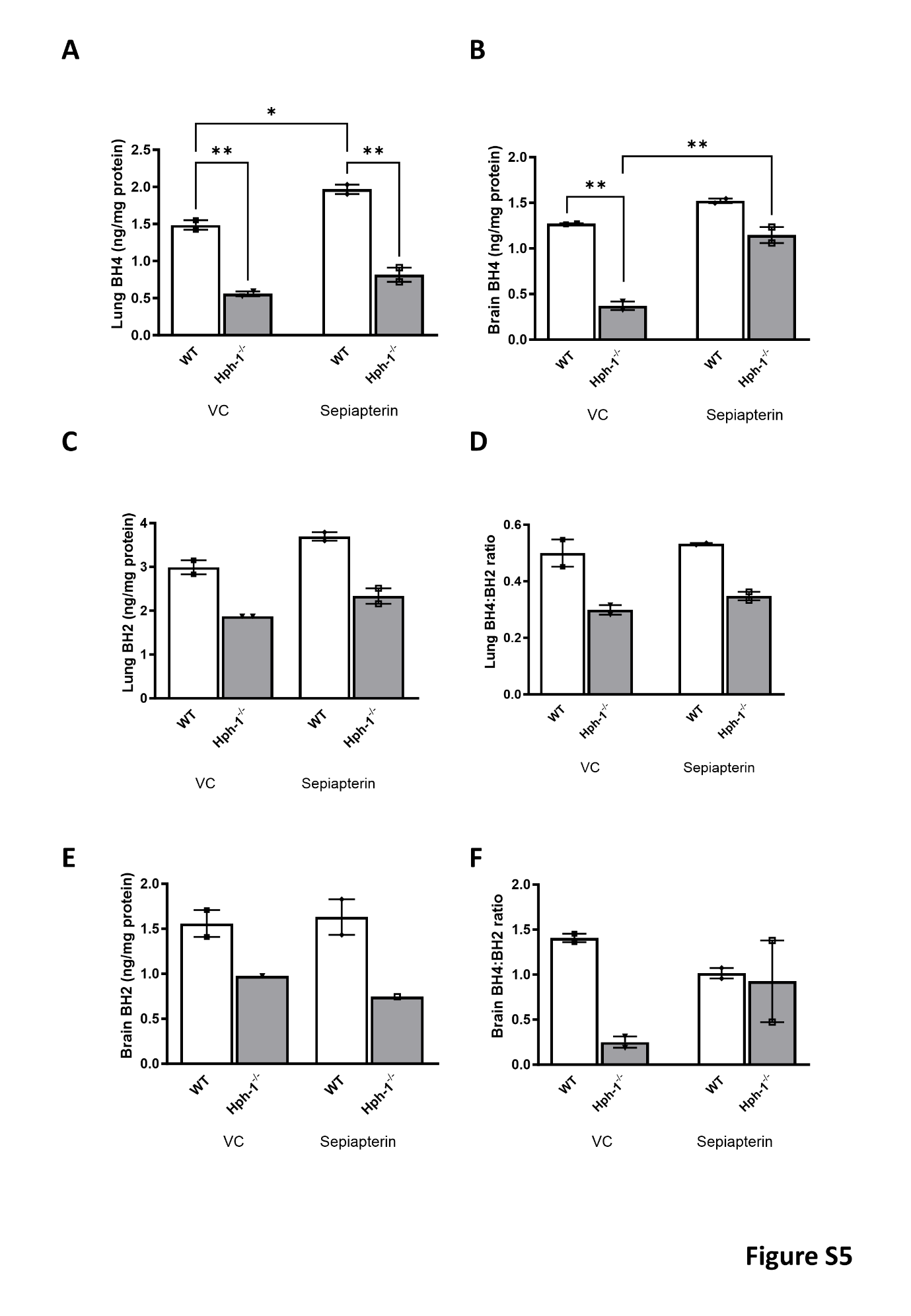
**

**
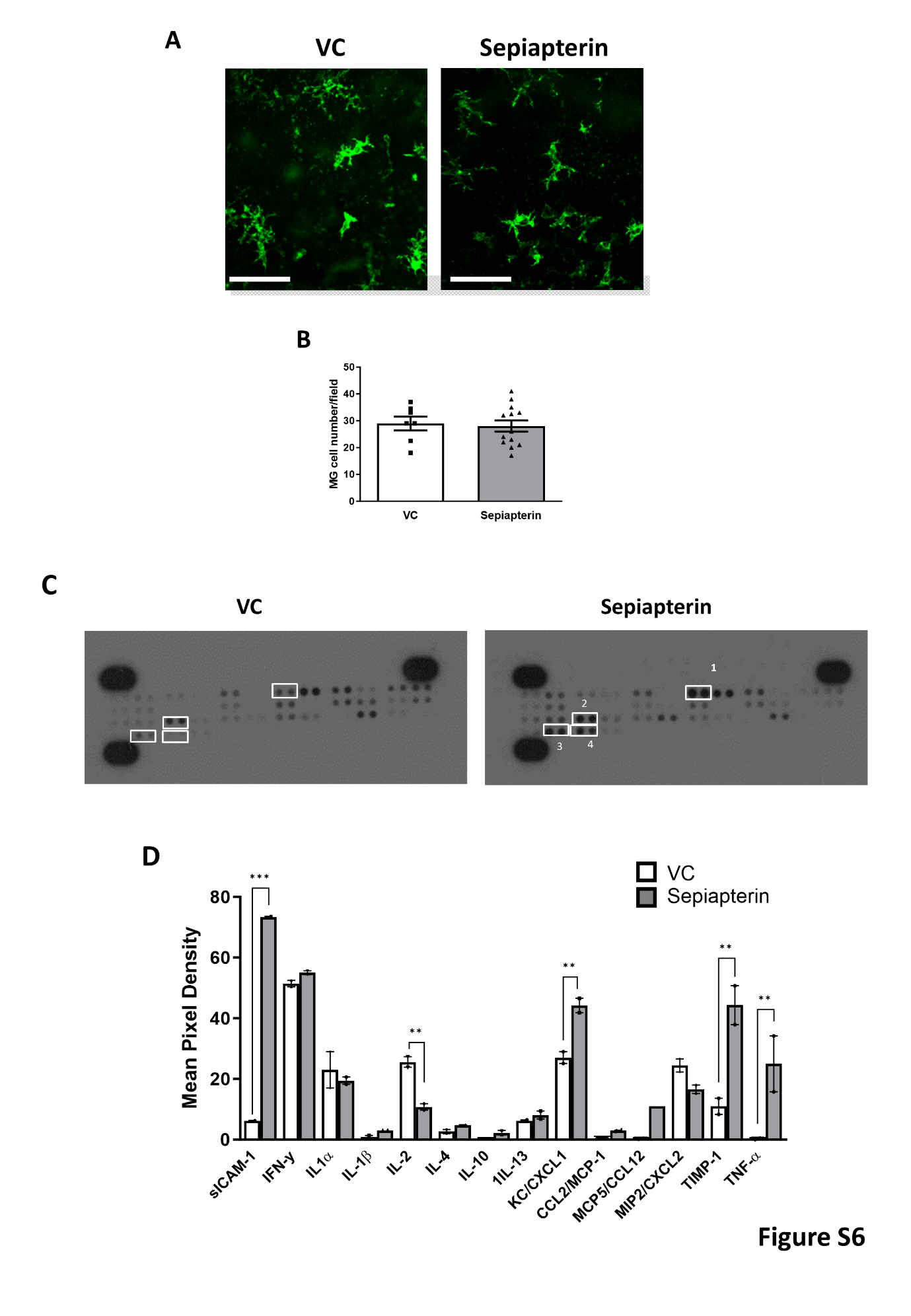
**

**
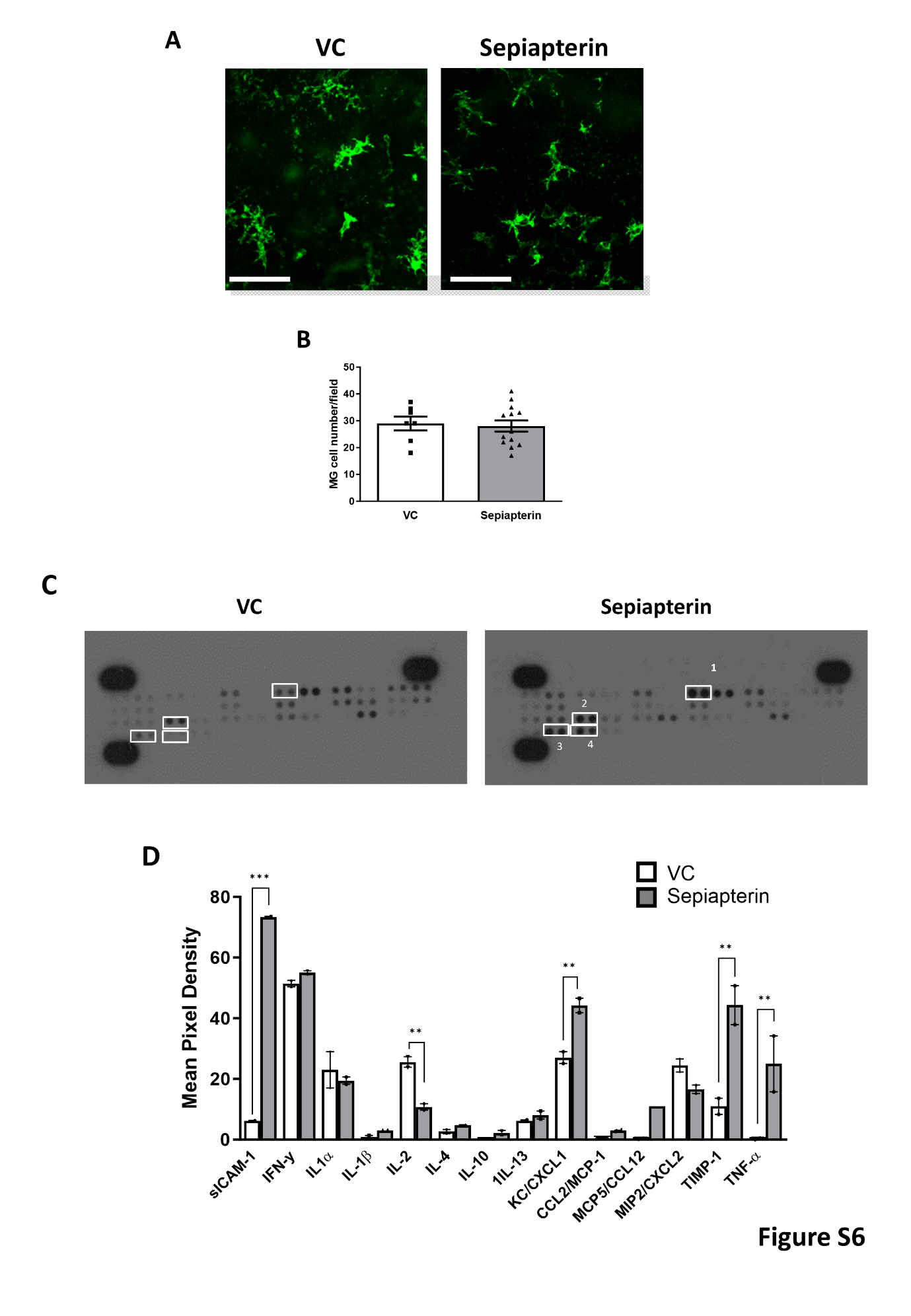
**

**
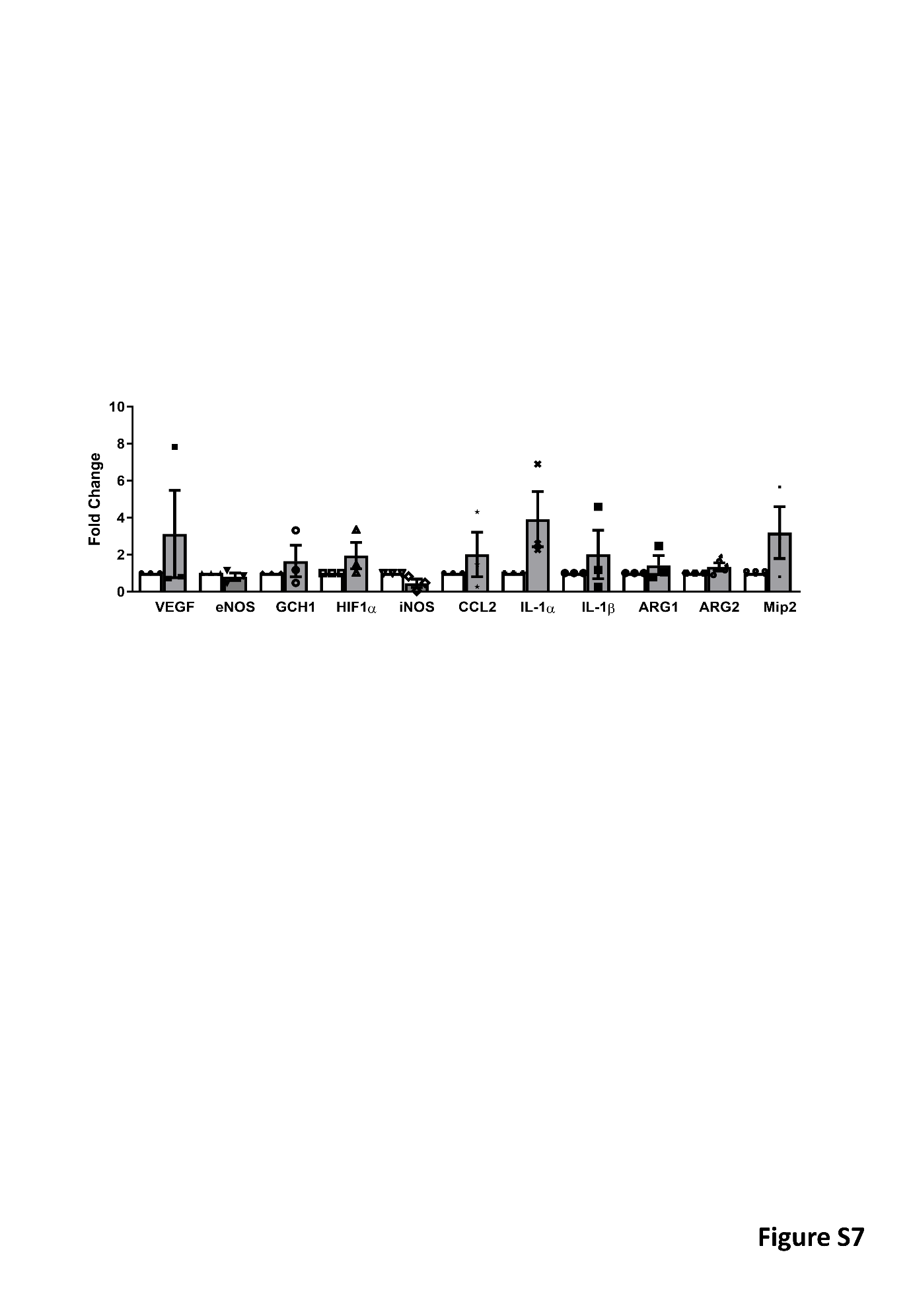
**

**Supplementary Table 1.** Mean data for all figures including heterozygote animals were used in experiments

|  | WT (mean ±SEM) | Heterozygote mutant hph-1^+/-^ | Homozygote mutant hph-1^-/-^ | Significance |
| --- | --- | --- | --- | --- |
| Figure 2B Avascular Area | 41.12 ± 1.025 | 36.30 ± 0.8733* | 35.93 ± 1.295* | * p< 0.05 vs WT (one way ANOVA with Bonferroni's Multiple Comparison Test) |
| Figure 2D TUNEL Fluorescence | 41.69 ± 3.673 | # | 29.39 ± 1.293* | * p=0.034 vs WT (unpaired t-test) |
| Figure 3A NOS Activity | 28.64 ± 1.733 | # | 20.51 ± 0.600* | * p=0.047 vs WT (unpaired t-test) |
| Figure 3C DHE Fluorescence | 1076 ± 183.5  894.1 ± 114.3  448.5 ± 123.0 | # | 693.6 ± 87.73*  670.2 ± 111.1  367.8 ± 75.93 | * p< 0.05 vs WT DHE (one way ANOVA with Bonferroni's Multiple Comparison Test) |
| Figure 3E NT Fluorescence | 272.6 ± 16.82 | # | 59.51 ± 4.644*** | ***p< 0.0001 vs WT (unpaired t-test) |
| Figure 4B MG cell number | 19.17 ± 0.7099 | 21.67 ± 1.154 | 24.10 ± 1.549* | * p< 0.05 vs WT (one way ANOVA with Bonferroni's Multiple Comparison Test) |
| Figure 5A NOS Activity | 66.93 ± 7.315  89.97 ± 10.97 (SEPSUP) | 58.69 ± 0.2500  66.17 ± 4.802 (SEPSUP) | 33.59 ± 10.56  50.78 ± 2.798 (SEPSUP)* | * p< 0.05 vs WT (one way ANOVA with Bonferroni's Multiple Comparison Test) |
| Figure 5B DHE Fluorescence | 2465 ± 52.54  1444 ± 119.9 (SEPSUP)+++ | 1821 ± 11.79*  1088 ± 33.00 (SEPSUP)¥¥ | 1648 ± 106.7**  924.8 ± 156.0 (SEPSUP)§§ | * p< 0.05, **p<0.01 vs WT (one way ANOVA with Bonferroni's Multiple Comparison Test)  +++ p< 0.001 vs WT (one way ANOVA with Bonferroni's Multiple Comparison Test)  ¥¥ p< 0.001 vs Hph-1^+/-^ (one way ANOVA with Bonferroni's Multiple Comparison Test)  §§ p< 0.001 vs Hph-1^-/-^ (one way ANOVA with Bonferroni's Multiple Comparison Test) |
| Figure 5C NT Fluorescence | 63.81 ± 7.171  28.05 ± 2.751 (SEPSUP)*** | 31.80 ± 3.544**  31.88 ± 3.404 (SEPSUP) | 27.10 ± 4.434***  24.66 ± 1.116 (SEPSUP) | * p< 0.05, **p<0.01, ***p<0.001 vs WT (one way ANOVA with Bonferroni's Multiple Comparison Test |
| Figure 6A TUNEL Fluorescence | 410.0 ± 25.07  187.8 ± 50.95 (SEPSUP)* | 212.3 ± 49.38  145.8 ± 14.59 (SEPSUP) | 147.7 ± 38.16*  118.0 ± 44.02 (SEPSUP) | * p< 0.05 vs WT (one way ANOVA with Bonferroni's Multiple Comparison Test) |
| Figure S1. Replicate data for NT immunofluorescence | 359.9 ± 20.14  137.6 ± 12.20 | # | 111.0 ± 9.453***  76.79 ± 5.718*** | ***p< 0.0001 vs WT (unpaired t-test)  ***p< 0.0001 vs WT (unpaired t-test) |
| Figure S3B Total TH coverage | 32.75 ± 7.181 | 21.00 ± 6.633 | 11.75 ± 2.136* | * p< 0.05 vs WT (one way ANOVA with Bonferroni's Multiple Comparison Test) |
| Figure S3B Total number of TH positive pericellular rings | 32.00 ± 4.041 | 24.00 ± 2.646 | 24.00 ± 4.000 |  |
| Figure S3C P7 Hph-1 qPCR data | mRNA change relative to 18S  GCH1 1.015 ± 0.1226  eNOS 1.002 ± 0.04  nNOS 1.018 ± 0.1218  TH 1.077 ± 0.3000  Notch1 1.169 ± 0.4090  Notch4 0.9990 ±0.02364  Dll4 1.186 ± 0.4764  Hey1 1.212 ± 0.5518  Sox17 1.048 ± 0.2115 | 0.5888 ± 0.007110  1.409 ± 0.08855  1.891± 0.4893  0.8549 ± 0.1262  1.107 ± 0.2386  0.9771 ± 0.1957  1.099 ± 0.4087  1.132 ± 0.3365  0.8244 ± 0.08335 | 0.1406 ± 0.05073***  0.654 ± 0.2448  0.5952 ± 0.1171  0.4505 ± 0.1173*  0.6404 ± 0.4403  0.3807 ± 0.08820 *  0.4771 ± 0.2268  0.7541 ± 0.2793  0.3311 ± 0.1187* | *** p< 0.001 vs WT (one way ANOVA with Bonferroni's Multiple Comparison Test)      * p< 0.05 vs WT (one way ANOVA with Bonferroni's Multiple Comparison Test)    * p< 0.05 vs WT (one way ANOVA with Bonferroni's Multiple Comparison Test)    * p< 0.05 vs WT (one way ANOVA with Bonferroni's Multiple Comparison Test) |
| Figure S4 P9 SEPSUP WT BH4 data | BH4  Retina- 1.594 ± 0.08012 (VC) 2.412 ± 0.1069 (Sepiapterin)  Brain- 1.067 ± 0.06242 (VC) 1.714 ± 0.0016 (Sepiapterin)  Lung- 1.548 ± 0.1025 (VC) 2.674 ± 0.087 (Sepiapterin)  BH2  Retina- 0.3864 ± 0.07263 (VC) 0.6027 ± 0.1692 (Sepiapterin)  Brain- 1.003 ± 0.08660 (VC) 1.086 ± 0.06686 (Sepiapterin)  Lung- 0.4503 ± 0.03066 (VC) 0.4815± 0.02331(Sepiapterin)  BH4:BH2 ratio  Retina- 5.695 ± 0.7318 (VC) 6.483 ± 0.6671 (Sepiapterin)  Brain- 1.565 ± 0.1299 (VC) 1.807 ± 0.1472 (Sepiapterin)  Lung- 6.178 ± 0.7769 (VC) 8.255 ± 0.5282(Sepiapterin) | ** p=0.0036 vs WT (unpaired t-test)  ** p=0.0092 vs WT (unpaired t-test)  * p=0.0139 vs WT (unpaired t-test) |  |  |
| Figure S5A BH4 (Lung) | 1.487 ± 0.0640  1.967 ± 0.0640 (SEPSUP) | 0.8788 ± 0.0640*  1.599 ± 0.112 (SEPSUP)++ | 0.5588 ± 0.0320**  0.8148 ± 0.0960 (SEPSUP)*** | * p< 0.05, **p<0.01, ***p<0.001 vs WT (one way ANOVA with Bonferroni's Multiple Comparison Test) ++ p< 0.01 vs Hph-1^+/-^ (one way ANOVA with Bonferroni's Multiple Comparison Test) |
| Figure S5B BH4 (Brain) | 1.270 ± 0.006318  1.522 ± 0.02480 (SEPSUP) | 0.4937 ± 0.02177  1.703 ± 0.3642 (SEPSUP)** | 0.3727 ± 0.04674  1.147 ± 0.08821 (SEPSUP)++ | **p<0.01, vs Hph-1^+/-^ (one way ANOVA with Bonferroni's Multiple Comparison Test) ++ p< 0.01 vs Hph-1^-/-^ (one way ANOVA with Bonferroni's Multiple Comparison Test) |
| Figure S5C BH2 (Lung) | 2.991 ± 0.1600  3.695 ± 0.0960 (SEPSUP) | 2.175 ± 0.01600  2.831 ± 0.3200 (SEPSUP) | 1.871 ± 0.0*  2.335 ± 0.1760 (SEPSUP)* | * p< 0.05 vs WT (one way ANOVA with Bonferroni's Multiple Comparison Test) |
| Figure S5D BH4:BH2 ratio (Lung) | 0.4997 ± 0.04813  0.5322 ± 0.003493 (SEPSUP) | 0.4043 ± 0.03240  0.5766 ± 0.1047 (SEPSUP) | 0.2987 ± 0.01710  0.3479 ± 0.01490 (SEPSUP) |  |
| Figure S5E BH2 (Brain) | 1.558 ± 0.1487  1.630 ± 0.1973 (SEPSUP) | 1.336 ± 0.02374  1.213 ± 0.02234 (SEPSUP) | 0.9766 ± 0.0  0.7450 ± 0.0 (SEPSUP) |  |
| Figure S5F BH4:BH2 ratio (Brain) | 1.408 ± 0.04737  1.016 ± 0.05831 (SEPSUP) | 0.3969 ± 0.02675  1.745 ± 0.4020 (SEPSUP) | 0.2496 ± 0.06279*  0.9262 ± 0.4529 (SEPSUP) | * p< 0.05 vs WT (one way ANOVA with Bonferroni's Multiple Comparison Test) |
| Figure S6B MG cell number |  | 29.00 ± 2.552  28.04 ± 2.110 (SEPSUP) |  |  |

# No experiment performed
